# Hybridizing slime mould algorithm with simulated annealing algorithm: a hybridized statistical approach for numerical and engineering design problems

**DOI:** 10.1007/s40747-022-00852-0

**Published:** 2022-09-21

**Authors:** Leela Kumari Ch, Vikram Kumar Kamboj, S. K. Bath

**Affiliations:** 1 Domain of Power Systems, School of Electronics and Electrical Engineering, Lovely Professional University, Punjab, India; 2 Department of Electrical and Software Engineering, Schulich School of Engineering, University of Calgary, Calgary, Canada; 3 Department of Electrical Engineering, GZSCCET MRSPTU Bathinda, Punjab, India

**Keywords:** CEC-2005, Hybrid search algorithms, Metaheuristics search, Engineering optimization

## Abstract

The existing slime mould algorithm clones the uniqueness of the phase of oscillation of slime mould conduct and exhibits slow convergence in local search space due to poor exploitation phase. This research work exhibits to discover the best solution for objective function by commingling slime mould algorithm and simulated annealing algorithm for better variation of parameters and named as hybridized slime mould algorithm–simulated annealing algorithm. The simulated annealing algorithm improves and accelerates the effectiveness of slime mould technique as well as assists to take off from the local optimum. To corroborate the worth and usefulness of the introduced strategy, nonconvex, nonlinear, and typical engineering design difficulties were analyzed for standard benchmarks and interdisciplinary engineering design concerns. The proposed technique version is used to evaluate six, five, five unimodal, multimodal and fixed-dimension benchmark functions, respectively, also including 11 kinds of interdisciplinary engineering design difficulties. The technique’s outcomes were compared to the results of other on-hand optimization methods, and the experimental results show that the suggested approach outperforms the other optimization techniques.

## Introduction

Nowadays, the usage of metaheuristic algorithms has become widespread in numerous applied fields because of their advanced presentation with less computing duration than other determinant algorithms in dissimilar optimization issues [1]. Uncomplicated conceptions are necessary to attain good outcomes, as well it is effortless to immigrate to dissimilar disciplines. In addition, the need for randomness in a while period of a few determinant algorithms prepares it leaning to go under local optima, and random parameters in metaheuristics make the algorithms explore for every optimum finding in search space, therefore, efficiently escaping local optimum. According to [2], the stochastic algorithms are less efficient than gradient descent algorithms for using gradient information. It is noticed that gradient descent algorithms have the convergence speed quicker than metaheuristic methods. On the other hand, a metaheuristics algorithm classically begins the optimization procedure at randomly produced outcomes and does not require gradient information, thus composing the algorithm extremely appropriate for realistic complications when the derivative data are not known. In reality, the solution location of several issues is repeatedly undetermined or endless. This might be impossible to bring out optimum solutions by bisecting the solution location over existing situations. Metaheuristics algorithms notice the immediate optimum solution of the issue by examining a huge solution space in random by sure means, to discover or produce enhanced solutions for the optimization issue over inadequate conditions or computational ability [3].

In context to the above discussions, an intermingle variant slime mould optimization algorithm was introduced using a simulated annealing technique into the planned research work, and the suggested hybrid translation of population-based metaheuristics search technique was examined to unravel the unique standard customary benchmark issues: unimodal, multimodal, fixed dimensions. Apart from this, the suggested optimizer’s performance was evaluated for engineering design and optimization problems for a more thorough investigation.

Earlier to this article, a few researchers introduced the same algorithm; however, the mode of idea of the algorithm and handling outline is quite diverse from the algorithms suggested in this article. A hybrid optimizer adaptive β hill climbing was combined with slime mould algorithm, here slime mould algorithm is in addition strengthened with Brownian motion and tournament selection to improve exploration capabilities thus producing better quality outcomes in the exploitation phase [4]. Zheng-Ming Gao et al. [5] introduced a technique named grey wolf optimizer–slime mould algorithm (GWO-SMA) to minimize the influence of uncertainty as low as probable which is best suited for a few benchmark functions and not suggested for engineering design issues. Juan Zhao et al. [6] proposed an improved slime mould algorithm with Levy flights and observed that when improved SMA is replaced with uniformly distributed factor, it performed well and when improved SMA is replaced with Gauss Distributed factor, it stuck in local minima. Juan et al. [7] promoted the Chaotic SMA–Chebyshev map and observed its performance to be a better, stable, more rapid, and better choice to apply in real engineering issues. Levy flight–slime mould (LF-SMA) algorithm [8] is clout by the actions of slime mould which is further mixed up with Levy distribution and excelled in obtaining better results in the exploration phase. Improved slime mould algorithm (ISMA) is developed from traditional SMA in two aspects: the exploration phase is aided with a modified opposition-based learning technique as well the phase of exploitative is aided with Nelder–Mead simplex search method to adjust the parameters of the controllers to control the velocity of a DC motor by a fractional-order PID (FOPID) controller as well as requires to maintain the automatic voltage regulator (AVR) at its level of terminal voltage through a PID in addition with second-order derivative (PIDD^2^) controller [9]. The combination of slime mould and Harris hawks optimization (HHO-SMA) implemented in [10] stood better technique to improve slow convergence speed. The improved version of slime mould with cosine controlling parameters in [11] could abolish errors as well as give better outcomes in engineering design issues. Accordingly, [12] finds the solution to a single solution optimization issue by replicating the 5 existence series of amoeba *Dictyostelium discoideum*: a phase of vegetative, aggregative, mound, slug, and dispersal using ε-ANN to build an initiative stage. To assess the Pareto optima solutions, the authors in [13] suggested multi-objective slime mould algorithm (MOSMA) for better convergence in association with elitist non-dominated sorting method. Considering two forms of slime mould arrangement [14] introduced a technique to build a wireless sensor setup to correlate two distinct local routing protocols. The network named Physarum is united with the ant colony model to enhance the technique’s proficiency to escape confined optimum values to treat the migrating agent issue efficiently [15]. Motivated by the dissemination of slime mould, Schmickland [16] suggested a bio-motivated navigation method framed for swarm robotics. Promotion of inexpensive and fault-tolerant charts based on foraging procedure of slime mould is done in [17]. According to above conversation, the majority of the pattern slime mould methods have been worn graph theory as well as in generation networks. Thus, the nature of this creature influenced scholars in the field of graph optimization [18]. Monismith et al. [12] simulated the life cycle of amoeba *Dictyostelium discoideum* to utilize the developed algorithm to optimize the issue with less experimental proofs.

With a unique pattern, a hybrid combination hSMA-SA introduced in this work primarily mimics the nature of slime mould’s foraging as well as morphological transforms. Meanwhile, the usage of adaptive weights in SMA simulates in producing positive and negative feedback throughout foraging, hence creating three diverse morphotypes. The simulated annealing algorithm is intermixed to boost the phase of exploitation of classical SMA resulting in improved results of the suggested hSMA-SA technique and proved better than already existing techniques. Overall, this approach is easier to use than prior population-based algorithms and requires fewer operators by least amount of computational struggles. The left behind parts of the current article enclose a literature review; background of suggested work in the next section; concepts of conventional slime mould algorithm (SMA), simulated annealing algorithm (SA), and the suggested hybrid hSMA-SA technique are discussed in the third section. The fourth section describes the standard benchmark functions. The fifth section displays the findings and compares them to those of other methods. In part 6, test on 11 engineering-based optimization design challenges has been carried, and the last section stands for the paper’s conclusion, limits, and future scope.

## Literature review

Single-based and population-based metaheuristics are two types of metaheuristic algorithms. According to the names addressed, the former case has only one solution in the whole optimization process, whereas in the latter case, a bunch of solutions is developed in every iteration of optimization. In population-based techniques, an optimal or suboptimal solution may be taken into consideration, which may be as similar as the optimal or precinct location. These population metaheuristics techniques frequently emulate nominal phenomena. These types of techniques usually start the procedure of optimization by developing a bunch of individuals (population), in which every individual of the population reflects an aspirant optimum solution. Accordingly, the population progress iteratively by making use of stochastic functions at times to reinstate the present population with a newly developed population. This procedure comes to an end unless and until the simulation process gets satisfied with end command.

Metaheuristics algorithms are naturally motivated by actual-world phenomenality to bring out improved solutions for optimization issues by resembling physical laws or biological experience. The physics-based techniques are those which applies mathematical conventions or techniques which includes sine cosine algorithm (SCA) [19], gravitational search algorithm (GSA) [20], central force optimization (CFO) [21, 22], charged system search (CSS) [23], and multi-verse optimizer (MVO) [24]. The two major classes of metaheuristic algorithms are evolutionary techniques and swarm intelligent methods which are nature-influenced strategies. The insight of the evolutionary algorithm emanates from the progression of biological fruition in environment, which when analyzed with conventional optimization algorithms; this is a global optimization technique having well robustness and appropriateness. The prevalent algorithms in the group of evolutionary algorithms are: genetic programming (GP) [25], evolutionary programming (EP) [26], biogeography-based optimization (BBO) [27] which helped in analyzing biological species geographical distribution, and explained how these can be utilized to infer techniques appropriate for optimization. Differential evolution (DE) [28] is an evolutionary algorithm which includes genetic algorithms, evolutionary strategies and evolution programming. Evolutionary programming (EP) and genetic algorithm (GA) [29] are haggard from Darwinian Theory and Evolution Strategy (ES) [30]. The purpose of EP, ES, and swarm-intelligence techniques in logical research, and real-time issues are wide-ranging rapidly [31].

**Fig. 1:**
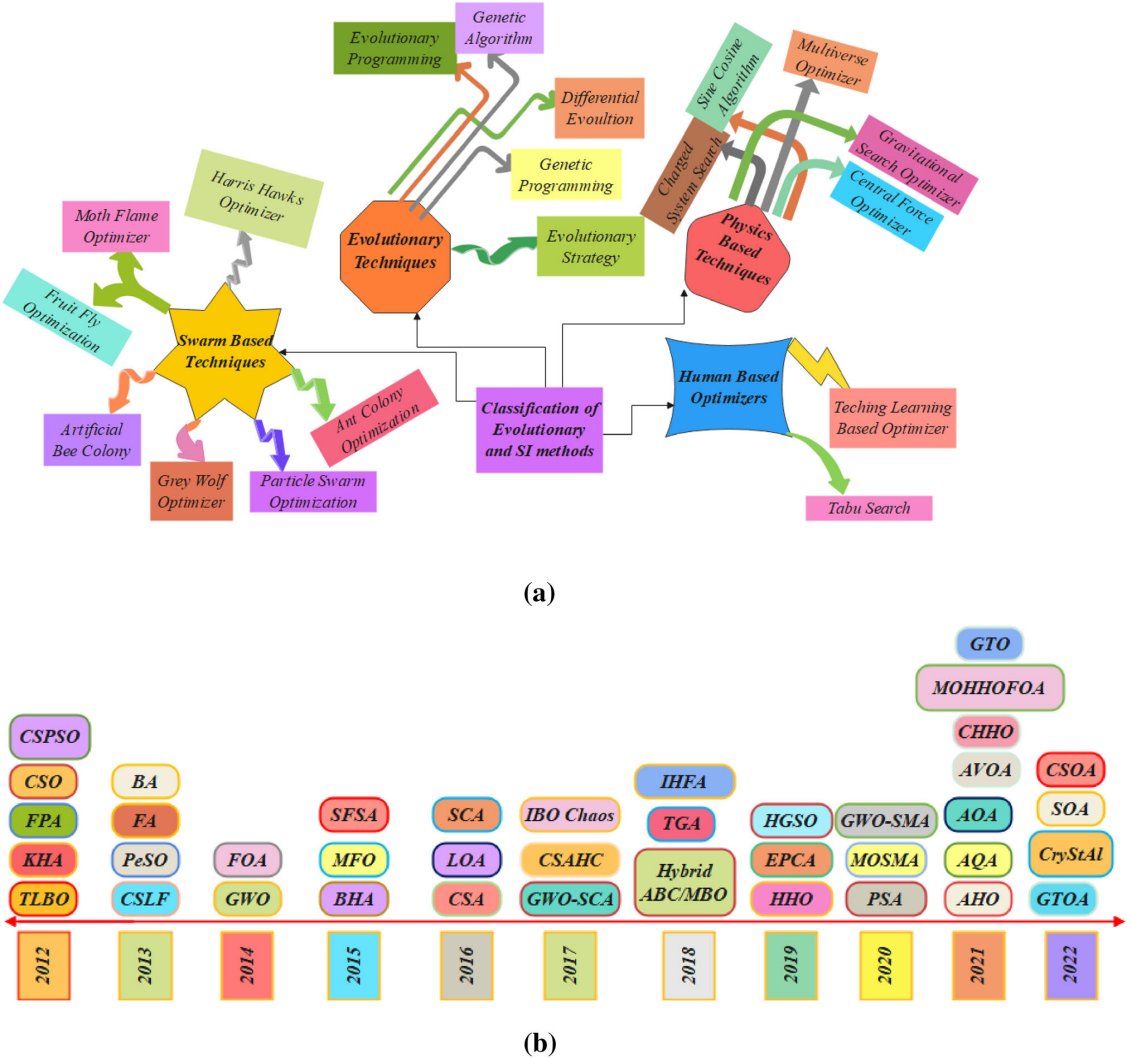
**a** Categories of SI and evolutionary methods. **b** Timeline of metaheuristics. *TLBO* teaching–learning-based optimization, *KH* krill herd, *FP* flower pollination, *CSO* cuckoo search algorithm, *CSPSO* cuckoo search particle swarm optimization, *CSLF* cuckoo search–Levy flight, *PeSO* penguins search optimization, *FA* firefly algorithm, *BA* bat algorithm, *GWO* grey wolf optimizer, *FOA* forest optimization algorithm, *BHA* black hole algorithm, *MFO* moth flame optimizer, *SFSA* stochastic fractal search algorithm, *CSA* crow search algorithm, *LOA* lion optimization algorithm, *SCA* sine cosine algorithm, *GWO-SCA* hybrid grey wolf optimizer and sine cosine algorithm, *CSAHC* cuckoo search algorithm with hill climbing, *IBO Chaos* improved butterfly algorithm with chaos, *Hybrid ABC/MBO* artificial bee colony with monarch butterfly optimization, *TGA* tree growth algorithm, *IHFA* improved hybrid firefly algorithm, *HHO* Harris hawks optimizer, *EPC* emperor penguins colony, *HGSO* Henry gas solubility optimization, *PSA* particle swarm optimization, *MOSMA* multi-objective slime mould algorithm, *GWO-SMA* hybrid grey wolf optimization–slime mould algorithm, *AHO* archerfish hunting optimizer, *AQ* Aquila optimizer, *AOA* arithmetic optimization algorithm, *AVOA* African vultures optimization algorithm, *CHHO* chaotic Harris hawks optimizer, *MOHHOFOA* multi-objective Harris hawks optimization fruit fly optimization algorithm, *GTO* gorilla troops optimization, *GTOA* modified group theory-based optimization algorithm, *CryStAl* crystal structure optimization, *SOA* seagull optimization algorithm, *CSOA* criminal search optimization algorithm

Swarm intelligence (SI) [32] encompasses a joint or communal intellect that unnaturally replicates the devolution of a biological bundle in the environment or the combined mannerisms of self-arranging structures. In this group of algorithms, the idea originates from biological communities present in the environment that have cooperative deeds and cleverness to accomplish an assured function. Reputable and current techniques in this set are particle swarm optimization (PSO) [33], moth flame optimization (MFO) [34], artificial bee colony (ABC) [35], Harris hawks optimizer (HHO) [36], fruit fly optimization algorithm (FFOA) [37], ant colony optimization (ACO) [38], and grey wolf optimization (GWO) [39]. Human-based techniques are those which resemble the activities of human works. In this group of algorithms, the inspiration starts from human activity in an assigned work that supports to finish the function assured. Teaching–learning-based optimization (TLBO) [40] imitates the teaching–learning procedure in a classroom, and tabu search (TS) [41]. A graphical diagram for the categorization of evolutionary and SI techniques is depicted in Fig. 1a and b displaying the history timeline of the other metaheuristic algorithms enclosed in this review. Table 1 showcases the last decade from the year (2012–2022) which exhibits the investigative works on finding solutions for numerical and engineering design problems.

**Table 1:** A look into population metaheuristics in a nutshell

Technique and its reference number	Name of the author and year	A quick summary
Seagull optimization algorithm (SOA) [55]	Yanhui Che and Dengxu He 2022	This paper proposed an enhanced seagull optimization algorithm to eliminate the defects of traditional seagull optimizer. The technique is tested on 12 various engineering optimization problems
Modified group theory-based optimization algorithms for numerical optimization (GTOA) [56]	Li et al. 2022	This paper concentrated on studying the applicability of the proposed GTOA to solve optimization problems by introducing two versions of GTOA which uses binary coding and integer coding. The performance proved to obtain better convergence rate and average accuracy
Criminal search optimization algorithm (CSOA) [57]	Srivastava et al. 2022	This paper introduced criminal search optimization algorithm which has been developed based on intelligence of policemen in catching a criminal. The presentation of the technique has been evaluated on standard benchmark functions—CEC-2005 and CEC-2020. Five test cases have been operated to measure the results of the suggested algorithm with other techniques and proved the good
Crystal structure optimization approach to problem-solving in mechanical engineering design (CryStAl) [58]	Babak Talatahari et al. 2022	The authors of this paper introduced a metaheuristic named crystal structure algorithm to discover solutions for engineering mechanics and design problems. Further, the technique has been examined on 20 benchmark mathematical functions and obtained satisfying outputs when measured with other existing methods
African vultures optimization algorithm (AVOA) [59]	Abdollahzadeh et al. 2021	The authors of this paper proposed African vultures optimization algorithm imitating the living style of African vultures foraging and navigation attitude. First, the method’s feat is tested on 36 benchmark functions and its applicability is announced on finding optimum solutions for 11 engineering design problems
Flow direction algorithm (FDA) [60]	Hojat Karami et al. 2021	This paper focused in proposing a physics-based algorithm named Flow direction algorithm imitating flow direction in a drainage basin. The method has been tested on 13, 10 and 5 classical mathematical, new mathematical benchmark functions and engineering design problems, respectively. These results proved better than other techniques results
A new hybrid chaotic atom search optimization based on tree-seed algorithm and Levy flight for solving optimization problems [61]	Saeid et al. 2021	The authors in this papers used combination of metaheuristic algorithms to crack 7 special engineering issues. This atom search algorithm convergence speed is enhanced by chaotic maps as well as Levy flight random walk. Furthermore, tree-seed method ties with ASO. These combinations of algorithms yield good results
A multi-objective optimization algorithm for feature selection problems (MOHHOFOA) [62]	Abdollahzadeh et al. 2021	The authors in this paper used three different solutions for feature selection. First, Harris hawks optimization algorithm is multiplied; second, fruit fly optimization algorithm is multiplied, and in third stage, these two algorithms have been hybridized to locate solutions for feature selection issues
Arithmetic optimization algorithm (AOA) [63]	Laith et al. 2021	This paper proposed arithmetic optimization algorithm and tested its performance on 29 benchmark functions 7 real-world engineering design problems. The outcomes obtained by this technique proved better among other existing methods
Aquila optimizer (AO) [64]	Laith Abualigah et al. 2021	The authors suggested population-based optimization method named Aquila optimizer to solve optimization problems. The technique has been evaluated on 23 benchmark functions and 7 real-life engineering design issues. The outcomes are good than other methods
Artificial gorilla troops optimizer (GTO) [65]	Abdollahzadeh et al. 2021	The authors in this paper proposed Artificial gorilla troops optimizer which is designed to improve the phases of exploration and exploitation. The algorithm is examined on 52 functions and 7 engineering design problems
Binary slime mould algorithm (BSMA) [66]	Abdel et al. 2021	This paper proposed slime mould algorithm with 4 binary versions for feature selection. All these versions were tested on 28 datasets of UCI repository
1D SMA models (SMAs) [67]	Sonia Marfia et al. 2021	This paper elevates the SMA 1D models to elucidate response of SMAs in thermo mechanical models
Slime mould algorithm (SMA) [68]	Davut Izci et al. 2021	Tested on several benchmark functions. Using PID controllers, the capability of SMA optimization is enhanced
Hybrid improved slime mould algorithm with adaptive β hill climbing (BTβSMA) [4]	Kangjian Sun et al. 2021	Tested on 16 benchmark functions and is suggested to lighten the unfledged global and local hunt in standard SMA
Archerfish hunting optimizer (AHO) [69]	Farouq Zitouni et al. 2021	Tested on 10 benchmark functions, 5 engineering problems. AHO replicates the behavior of Archerfish like jumping and shooting to find closer optimum values
WLSSA [70]	Hao Ren et al. 2021	Tested on 23 benchmark functions. With the combination of slap swarm and weight adaptive Levy flight have noticed finer optimum values
Multi-temperature simulated annealing algorithm (MTSA) [71]	Shih-Wei-Lin et al. 2021	This algorithm is developed to reduce the scheduling issues which influence the design and optimization of automated systems
Self-adaptive salp swarm algorithm (SASSA) [72]	Rohith Salgotra et al. 2021	Salp swarm algorithm is improved to mould it into self-adaptive by supplementing it by four modifications which possess in improving local search
Simulated annealing with Gaussian mutation and distortion equalization algorithm (SAGMDE) [73]	Julian Lee et al. 2020	This combined algorithm applied on different data sets yields better results in exploratory phase when only simulated annealing algorithm was applied
Slime mould algorithm (SMA) [3]	Shimin Li et al. 2020	Tested on 33 benchmark functions It replicated the characteristics of slime mould. SMA, intended to give better exploration capability and extending its application in kernel extreme learning machine
Hybrid grey wolf optimization–slime mould algorithm (GWO-SMA) [5]	Zheng-Ming Gao et al. 2020	Made 3 types of experiments resulting it did not give better results in combining GWO and SMA. SMA equations were unique and excellent and firm to progress
Chaotic SMA–Chebyshev map [7]	Juan Zhao et al. 2020	Tested on standard benchmark functions and noticed the results to be better and the technique performed faster with stability
Improved slime mould algorithm with Levy flight [6]	Juan Zhao et al. 2020	Worked to reduce the pressure of randomness and noticed SMA–Levy flight with uniform distributed parameters would give better results
Modified slime mould algorithm via Levy flight [8]	Zhesen Cui et al. 2020	Tested on 13 benchmark functions and 1 engineering design issue and notice the results obtained were better and steady
Hybridized Harris hawks optimization and slime mould algorithm (HHO-SMA) [10]	Juan Zhao et al. 2020	The research attentively made efforts on many updating discipline mostly individuals on swarms
Improved slime mould algorithm with cosine controlling parameter [11]	Zheng-Ming Gao et al. 2020	The research helped in finding that the controlling parameters are very essential for the technique to perform better, at the same time noticed that all parameters were not acceptably helpful. Hence, should find more apt method
Multi-objective slime mould algorithm based on elitist non-dominated sorting (MOSMA) [13]	Manoharan Premkumar et al. 2020	Tested on 41 various cases, constrained, unconstrained as well as on real-life engineering issues. On applying this algorithm resulted in high-quality and effectiveness solutions for tough multi-objective issues
PSA: a photon search algorithm [74]	Y. Liu and Li 2020	23 functions were put to the test. The characteristics of photons in physics were the inspiration for this piece. The algorithm has strong global search and convergence capabilities
Movable damped wave algorithm [75]	Rizk et al. 2019	This paper proposed movable damped wave algorithm and the algorithm has been examined on 23 benchmark functions and 3 engineering design problems
Henry gas solubility optimization: a novel physics-based algorithm (HGSO) [76]	Hashim et al. 2019	47 benchmark functions were used in the testing. It is modeled after Henry’s reign. HGSO, which aims to meet the check room and halt optima locale’s production and conservation capacities
Emperor penguins colony (EPC) [77]	Sasan et al. 2019	A new metaheuristic algorithm named emperor penguins colony is proposed in this paper and has been tested on 10 benchmark functions
Harris hawks optimization (HHO) [78]	Heidari et al. 2019	There were 29 benchmarks and 6 technical issues which were tested on. It is being introduced to help with various optimization chores. Nature’s cooperative behaviors, as well as the patterns of predatory birds hunting Harris’ hawks, impact the strategy
Tree growth algorithm (TGA) [79]	Armin et al. 2018	The authors introduced Tree growth algorithm which is inspired by trees competition for acquiring light and food. It has been examined on 30 benchmark functions and 5 engineering design problems
Hybrid artificial bee colony with monarch butterfly optimization [80]	Waheed et al. 2018	This paper introduced a new algorithm named hybrid ABC/MBO (HAM) and evaluated on 13 benchmark functions and proved better in outcomes
An improved hybrid firefly algorithm for solving optimization problems (IHFA) [81]	Fazli Wahid et al. 2018	This paper introduced a novel method called GA-FA-PS algorithm and tested on 3 benchmark functions and proved that the obtained results are better than firefly algorithm and genetic algorithm
An improved butterfly optimization algorithm with chaos [82]	Sankalap Arora et al. 2017	The authors in this paper improved butterfly optimization with chaos to increase its performance to avoid local optimum and convergence speed. The suggested chaotic BOAs are validated 3 benchmark functions and 3 engineering design problems
Cuckoo search algorithm–hill climbing technique (CSAHC) [83]	Shehab et al. 2017	The authors proposed new cuckoo search algorithm by hybridizing with hill climbing technique to solve optimization issues. It has been examined on 13 benchmark functions and proved successful
Hybrid GWO-SCA [84]	Singh et al. 2017	This paper proposed hybrid grey wolf optimizer and sine cosine technique and tested on 22 functions, 5 bio-medical dataset and 1 sine dataset problems
SCA: a sine cosine algorithm for solving optimization problems [85]	Seyedali Mirjalili 2016	The author proposed SCA for the solutions of optimization problems and its efficiency is validated on testing 19 benchmark functions
Lion optimization algorithm (LOA): a nature-inspired metaheuristic algorithm [86]	Maziar Yazdani et al. 2016	This paper introduced Lion optimization algorithm and has been examined on 30 benchmark functions
Crow search algorithm (CSA) [87]	Alireza Askarzadeh 2016	The author proposed crow search algorithm and applied to unravel 6 engineering design issues. The outputs were promising than existing methods
Stochastic fractal search: a powerful metaheuristic algorithm (SFS) [88]	Salimi 2015	Uni, multi, fixed functions, and engineering functions were all tested
Moth flame optimization algorithm: a novel nature-inspired heuristic paradigm (MFO) [34]	Mirjalili 2015	7 engineering designs were tested, as well as 29 benchmarks. This optimizer followed navigation tactic of moth flame. The outcomes of this method stood better than existing techniques
Solving optimization problems using black hole algorithm (BHA) [89]	Masoum Farahmandian et al. 2015	This paper suggested black hole algorithm and has been checked on 19 benchmark functions. These results were better than PSO and GA
Forest optimization algorithm FOA [90]	Ghaemi et al. 2014	This technique is for determining the utmost as well as minimum value using a practical appliance, as well as demonstrating that the FOA can generally solve that are acceptable
Grey wolf optimizer (GWO) [39]	Mirjalili, Mirjalili, and Lewis 2014	The researchers looked at 29 BFs and 3 optimization engineering-based approaches. The image was enthused by a swarm-intelligence optimization and was inspired by grey wolves. Grey wolves’ communal structure and hunting conduct were used to develop the suggested model
Cuckoo search algorithm using Lèvy flight: a review [91]	Sangita Roy et al. 2013	The authors in this paper discussed about cuckoo search algorithm using Levy flight algorithm and noticed that the presentation of this method is superior to particle swarm optimizer and genetic algorithm when examined on 10 benchmark functions
Firefly algorithm: recent advances and applications (FA) [92]	Xin-She Yang et al. 2013	This paper suggested firefly algorithm, its fundamentals and explained the balancing of exploration and exploitation phases. In addition, the technique has been tested on higher-dimensional optimization problems
Bat algorithm: literature review and applications (BA) [93]	Xin-She 2013	The author presented the literature review and applications of Bat methodology which is efficient for solving optimization issues
Penguins search optimization algorithm (PeSOA) [94]	Youcef Gheraibia et al. 2013	This paper presented penguins search optimization algorithm and tested on 3 benchmark functions and obtained better results
Teaching–learning-based optimization (TLBO) [40]	Rao et al. 2012	In a power system, TLBO has two stages: a teaching stage and a student stage. Interacting by way of both is feasible only via modification, and the issue is solved
Krill herd (KH) [95]	A. H. Gandomi et al. 2012	This paper proposed krill herd algorithm which is a biologically inspired algorithm. Tested on several benchmark functions
Flower pollination algorithm (FPA) [96]	Xin-She Yang 2012	The author in this paper proposed flower pollination method which is motivated by the procedure of pollination in flowers. The technique has been tested on 10 benchmark functions and 1 nonlinear design problem. The results were better than PSO and GA methods
A hybrid CS/PSO algorithm for global optimization [97]	Ghodrati et al. 2012	The authors in this paper presented hybrid CS/PSO method to crack optimization issues. The technique has been examined on many benchmark functions to prove better than other techniques
Biogeography-based optimization (BBO) [27]	Simon 2008	14 typical benchmark functions were used in the testing. The BBO method, which analyses the spatial distribution of biological species, may be used to derive optimization algorithms
A new heuristic optimization technique: harmony search (HS) [98]	Geem, Kim, and Loganathan 2001	The comparison of the music creation cycle inspired this algorithm. The starting values of the variables may not be required for HS to make a decision
Differential evolution (DE) [99]	Storn et al. 1997	It shows how to minimize nonlinear and non differentiable continuous space functions that are possibly nonlinear. It merely needs a few strong control variables drawn from a predetermined numerical range
Tabu search-part I (TS) [100]	Fred Glover 1989	This has originated as a method of resolving combinatorial real-world scheduling and covering challenges

Though different types of metaheuristics algorithms have some dissimilarities, two same phases namely explorative as well as exploitative match them in search phase progression [42]. This explorative stage specifies a procedure to discover solution location as broadly, arbitrarily, and globally as feasible. The exploitative stage specifies the proficiency of the technique to search further exactly in the arena promoted by the exploration stage and its arbitrariness reduces whereas accuracy rises. While the exploration capacity of the technique is leading, it hunts the solution location at random to generate extra discriminated answers to mingle hastily. When the technique’s exploitative capacity is dominant, it performs additional checks in local in such a way that the quality as well as accuracy of the results is enhanced. Moreover, while the exploration competence is enhanced, it reduces exploitation capability, and contrarily. One more defy is that the stableness of these two abilities need not be similar for dissimilar issues. Consequently, it is comparatively challenging to achieve a suitable balance among the two aspects that are proficient for all optimization issues.

Regardless of the victory of traditional and current metaheuristic algorithms, no other techniques can assure to discover the global optima for all optimization issues. No free-lunch (NFL) theory proved it sensibly [43]. Despite this, no one optimization technique has up till now been revealed to crack each and every optimization issues [44]. This theory provoked several researchers to propose new algorithms and find efficient solutions for new classes of issues. Huang et al. [45] made a blend of the crow search and particle swarm optimization algorithms. The spotted hyena optimizer (SHO) [46] is a revolutionary metaheuristic approach encouraged by spotted hyenas’ original combined actions in hunting, circling, and attacking prey. The whale optimization approach (WOA) [47] is a intermix metaheuristics approach that uses whale and swarm human-based optimizers to find optimal exploration and convergence capabilities. MOSHO [48] is a multi-objective spotted hyena optimizer that lowers several key functions. Hu et al. [49] rely on butterfly research into how they build scent as they migrate from one food source to another by the modified adaptive butterfly optimization algorithm (BOA). Balakrishna et al. [50] applied a metaheuristic optimization method HHO-PS that was created to identify a latest edition of Harris hawks for local and global search. The binary spotted hyena optimizer (BSHO) [51] is a discrete optimization problem-solving metaheuristic approach based on spotted hyena hunting behavior. The Bernstrain-search differential evolution method (EBSD) [52] is a universal differential evolution algorithm that depends on mutation and crossover operators that was suggested. The reliability-based design optimization method (RBDO) [53] addresses issues such as global convergence and intricate design variables. Ayani Nandi et al. [54] coupled Harris hawks’ virtuous behavior with arithmetic conceptions of sine and cosine to strengthen the abilities of the hybrid Harris hawks–sine cosine method (HHO-SCA) in the phases of exploration and exploitation.

### Background of suggested work

Nature has many organisms and every organism has a unique behavior; among them, few organisms behaviors will attract and can be straightforwardly adopted and statistically shaped to tackle nonconvex and unconstrained models. This adaptability made several researchers seek to imitate the operational procedure for computational and algorithms’ evolution. Based on this idea, slime moulds were accredited for the past few years. Slime mould (a fungus) lives in chill and muggy places stretching its venous to reach the food places. In the process of repositioning, a fan-shaped structure in the front side is formed and connected by a tail shape which acts as interconnection permitting cytoplasm to flow inside. Slime moulds make use of venous structures in search of various food points which trap food places and creeps very eagerly, if there is a scarcity of food, which aid to recognize their behavior in searching, moving, and catching the food in the varying environment. Slime mould is good enough to adjust positive and negative feedbacks depending on the distance to catch the food in an improved way proving that it pay a predefined aisle to reach the level of a food source by all the time targeting rich food spots and based on food stock as well as environmental changes. The slime mould counterbalances the speed and elects to leave that region, and starts its fresh search before foraging. Slime mould decides to search new food centers based on the data available and leaves the current region during foraging. Slime mould is also clever enough to divide its biomass to other resources to grasp enrich food even though it has abundant foodstuff currently. It adjusts as per the foodstuff available in reaching the target. Despite having good global search capability, slime mould lacks in local search ability and convergence. To enhance local search aptitude and convergence speed, the slime mould method in this article is combined with simulated annealing which is good at local search. The recommended calculation aims to increase the convergence rate and betterment in local search of slime mould algorithm utilizing simulated annealing; thus, hSMA-SA is introduced.

The researchers pursue motivation from the streams of physics, genetics, environment, and sociology to develop a state-of-the-art metaheuristic algorithm. In the suggested work, the authors sought to solve these issues by heuristically mixing two strong algorithms for improved exploration and exploitation, as well as enhanced search capabilities. The following research papers were picked from already available techniques which were taking much time to reach near global minima and increased computational burden: animal migration optimization (AMO) algorithm [101], sine cosine algorithm (SCA) [102], group search optimizer (GSO) algorithm [103], interior search algorithm (ISA) [104], electro search optimization (ESO) algorithm [105], tunicate swarm algorithm (TSA) [106], orthogonally designed adapted grasshopper optimization (ODAGO) algorithm [107], photon search algorithm (PSA) [74], gradient-based optimizer (GBO) [108], transient search optimizer (TSO) [109], dynamic group-based cooperative optimizer (DGBO) [110], central force optimization (CFO) algorithm [21], electromagnetic field optimization (EFO) algorithm [111], harmony search algorithm (HS) [98]. Few such hybrid algorithms are manta ray foraging optimization (MRFO) algorithm [112], life choice-based optimizer (LCBO) [113], improved fitness-dependent optimizer algorithm (IFDO) [114], incremental grey wolf optimizer, and expanded grey wolf optimizer (I-GWO and Ex-GWO) [115], hybrid crossover oriented PSO and GWO (HC-PSOGWO) [116], self-adaptive differential artificial bee colony (SA-DABC) [117], and multi-objective heat transfer search algorithm (MHTSA) [118].

The remaining part of this section of the paper is contributed to describe the latest survey on SMA variants and SA variants, novelty of the proposed technique and background of suggested work.

### Literature survey on slime mould algorithm variants and simulated annealing algorithm variants

In this area, a special relevant study has been offered to discover data on current advancements connected to SMA variants, as well as newly created approaches by various researchers. By imitating the behavior of slime mould in discovering food points, the researchers have developed a broad assortment of metaheuristic and hybrid renditions of SMA to tackle many types of stochastic problems, as evidenced by the cited literature studies. Using a heuristic method, a group of academics was assessed to examine real-time issues, namely network foraging, engineering design, image segmentation, optimum power flow, structural machines, fault-tolerant transportation, and feature selection are the topics covered. The correctness of any algorithm’s answer is determined by its ability to strike a proper balance between intensification and variety. Slow convergence is a frequent issue with many heuristic techniques, noticed according to studies. The computing efficiency falls as a result. As a result, hybrid algorithms are becoming increasingly popular for improving solutions effectively. Many researchers have successfully used various SMA methods to optimize particular key functions. The eventual goal of these techniques is to find the best answer to an issue. Researchers have newly formed novel SMA renditions for a diversity of operations: chaotic slime mould algorithm (CSMA) [119]; here, the sinusoidal chaotic function is merged with traditional SMA to improve the exploitation capability of SMA. Hybrid arithmetic optimizer–slime mould algorithm (HAOASMA) [120] is introduced to solve the less internal memory and slow convergence rate at local optimum by repeatedly exciting arithmetic optimizer with slime mould algorithm and vice versa which improves the population to skyrocket the convergence. Slime mould algorithm with Levy flights (SMALF) [6] is introduced to enhance searching ability by replacing random weights with Levy flights. OBLSMAL [121] method was proposed by adding two search techniques to basic SMA, i.e., initially, an opposition-based learning algorithm has been utilized to boost up the rate of convergence of SMA, and later SMA is assisted with Levy flight distribution to improve exploration and exploitation phases. Thus, OBLSMAL proved better in convergence rate and searching tactics than other algorithms. Hybrid slime mould salp swarm algorithm (HSMSSA) [122] is developed to improve the convergence speed and searching abilities. A successful method LSMA [123] is suggested in terms of both multilayer threshold precision and time. For both discovery and exploitation, it is necessary to have features of decreased calculations.

Some variants of simulated annealing are: simulated annealing with adaptive neighborhood search algorithm (SA-ANS) [124], developed to find solutions when the algorithm is stuck in the same solution, i.e., at every iteration, more number of solutions are found. Harris hawks optimization with simulated annealing [125] is used for feature selection, as the SA algorithm is added up with HHO attains a reduction in consuming time, this novel idea finds a solution for complex optimization problems in CT-scan in detecting COVID-19. To reduce the high time complexity of capacitated vehicle routing issues, an enhanced simulated annealing algorithm combined with crossover operator (ISA-CO) was suggested in [126] to improve convergence. Using hidden Markov model (HHM), dynamic simulated annealing was introduced in [127], with the integration of HHM adapts neighborhood structure at every iteration in SA, thus proving the capability optimum nature of fellow function depending on the history of search. On the whole, in every observation of an algorithm, it is noted that many cases experience precipitate convergence in simulation results.

The introduced Lévy flight distribution and simulated annealing algorithm (LFDSA) [128] involves a balanced structure in both the phases of exploration and exploitation and proved excellent by testing on unimodal, multimodal benchmark functions and non-parametric statistical tests. This enhanced capability of the suggested algorithm helped to achieve optimum values of fractional-order proportional-integral derivative (FOPID) parameters for an improved closed-loop output voltage control performance of the buck converter in terms of time and frequency domain reaction as well as disturbance rejection. Considering a single machine infinite bus power system, the improved atom search optimization algorithm (IASO) [129], a recently developed hybrid approach that was built by integrating atom search optimization and simulated annealing approaches, is utilized to optimize a power system stabilizer function. In this study, the improved approach was used to find optimal controller settings for a power system stabilizer damping controller, proving the potential and improved feat of the recommended method for a difficult practical engineering problem. On comparison of outcomes with other algorithms, the proposed technique stood better. Atom search algorithm with simulated annealing (hASO-SA) [130] is used to answer various optimization issues, as the simulated annealing technique assists the ASO to avoid local minima and also helps to raise the level of diversity in search of optimal solution in the search space. This mixture version of algorithm (hASO-SA) feat the fast-optimum searching ability and hill climbing act of both ASO and AS techniques which adds the aptitude of the suggested algorithm to solve different optimization issues. Later hASO-SA is applied in training MLP in three diverse techniques using metaheuristic techniques. The first technique is involved to discover linked weights and biases which help to achieve reduced error for an MLP. The second technique is to discover a suitable structural design for an MLP to handle a specific problem using metaheuristics. In the third method, the parameters such as learning rate of the gradient-based learning algorithm and momentum are tuned.

As it is also well known, the burning issue is the struggle of finding answers to optimization problems. The difficulty of optimization problems will expand as the number of optimization factors grows. Furthermore, several of the planned deterministic methods are prone to local optima trapping and have a slow convergence rate. Metaheuristic nature-motivated optimization methods are utilized to tackle such issues. Two major characteristics of these methods are the lack of beginning presumptions and population dependence. There has yet to be identified an optimization method that can address all optimization issues [44]. This motivated to launch the slime mould–simulated annealing algorithm, a metaheuristic hybrid variation optimizer (hSMA-SA).

In the following three ways, the newly proposed hybridized SMA variant outperforms numerous population-based metaheuristic techniques.

The first step comprises combining two established procedures to develop a trouble-free and proficient simulation method that, when compared to other current methods, does more complex mathematical computations faster. Standard SMA features are introduced as initial parameters into the SA technique to increase its progressing capacity and to optimize these values in order to improve standard SMA’s ability to assess the ideal value of an optimization trouble. This treatment is completed without the use of complex procedures.

The second point is that in terms of results, the suggested new method outperformed the classic SMA solution. In the outcome section, the empirical outputs serve as proof, confirming its numerical and experimental performance. This sets the suggested approaches apart from other methods. Most techniques fail to find an optimal solution with an increasing number of repetitions because to inherent limits. The proposed technique provides an essential and standard strategy to manage this issue by assessing the operational phases of this approach, which may be used by other optimization approaches.

The hSMA-SA technique’s third aspect is that it aims to increase the optimization strength of traditional SMA in order to obtain optimal values while keeping the algorithm's complexity low. Combining the SA algorithm with the regular SMA yields the proposed optimization approach. Each of the two mathematical models discussed above has its own framework for dealing with optimization. To convert the ideology of one algorithm into the principles of another, computational methodologies are applied. As a consequence, in this work, the SMA oscillation mode is mapped into SA parameters, and the SA features are translated back into SMA. To raise the complexity of hybrid variations, latest operators have been suggested to this approach. Sixteen benchmark functions along with 11 special engineering optimum problems are investigated to evaluate the proposed hybrid version hSMA-SA with various parameter choices. The results outperform those of other algorithms currently in use. The subsequent are the most important things in terms of new contribution:

(i) The simulated annealing algorithm is applied to advance the local search ability of SMA.
(ii) The SA approach has boosted the prominence of the preliminary population.
(iii) In order to preserve the uniqueness of SMA, parameters of SMA are untouched.
(iv) The hSMA-SA strategy has been profitably tested for 6 standard unimodal, 5 standard multimodal, 5 customary fixed-dimension benchmark functions, and 11 forms of multidisciplinary engineering design difficulties to test its effectiveness.
(v) The success of the new launched technique’s examination is done by Wilcoxon rank test.
(vi) As per the findings section’s comparison analysis, the suggested approach performed excellently the fitness evaluation in addition to solution precision.

## Proposed hybridized slime mould algorithm-simulated annealing algorithm

To assure a proficient algorithm, this research work suggested a new hybrid combination of metaheuristic algorithm named hybridized slime mould-simulated annealing algorithm. As the top conversation in the introduction of this paper, this method has been initiated depending on the dispersion and foraging behavior of slime mould. Mathematically, the structure of the propagation wave is represented in the discovery of a better approach to relate foodstuff with brilliant exploratory capability and exploitation affinity.

The traditional SMA is hybridized with simulated annealing algorithm to additionally improve the performance. Every time a new method arouses because of a few drawbacks which do not satisfy in solving many difficult optimization issues in which mathematical reformulations limit the efficiency of methods. The suggested method is beneficial than other algorithms including conventional SMA and SA algorithms. It is noticed that because of early convergence, the convergence rate is not proficient. The suggested algorithm uses the simulated annealing algorithm to enhance local search ability and improve the convergence of a traditional SMA and find a solution for various problems as well optimize the key fitness of those issues. The simulated annealing algorithm makes SMA adjust the starting parameters of the hunt and hence avoids the local trapping of slime moulds. It is well known that few methods are weak in global search. Every algorithm has the necessity to maintain equal balance among local and global search to obtain a proficient performance. In the suggested work, no complex operators are utilized to balance local and global search requirements. The computational time is less for the results drawn from the simulation process. In addition, trapping in local optimum is absent in the suggested method.

### Slime mould algorithm

**Fig. 2:**
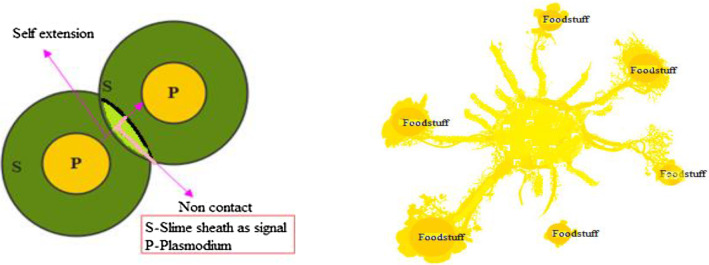
Growing crops slime mould morphology

Physarum polycephalum is the technical title for slime mould. In a 1931 article [131], Howard recognized it as a fungus, studied its span of life and named as “slime mould”. Slime mould grows and lives in cool and moist places. Plasmodium, the competent and active phase of slime mould, materializes to be its essential feeding phase. During this phase, the slime mould’s organic component looks for victuals, catches it, and produces enzymes to consume it. As depicted in Fig. 2, at the time of repositioning, a fan format is framed on the extension of the front end, and this fan format is escorted to permit cytoplasm to flow through it by an integrated venous network [132]. With their unique venous network, slime moulds search various food sources and consequently stash enzymes to grab the food points. Depending on the food availability in the environment, slime mould matures over 900 m^2^ [131].

Slime mould is referred to as a model organism [133] for the reason of its elegant quality for eating on agar and oatmeal. Kamiya et al. [134] looked at the cytoplasm flow of a slime mould in great detail, which cooperated them better understand the slime mould’s ability to obtain sustenance from its surroundings. As the vein reaches a foodstuff supply, the bio-oscillator sends out a propagation wave, which speeds up cytoplasm stream contained by the vein [135]. The quicker the cytoplasm flows, the stronger the vein turns. The slime may build out its optimum path to gather the victuals in a silent better mode using a combination of positive and negative feedback. As a result, the mathematical representation for use in graph theory and route networks [136] was slime mould.

The venous arrangement increases phase variety in the contraction mode in slime mould [135], leading to the discovery of three relationships between morphological changes in the venous configuration and slime mould contraction phase.

(i) As the contraction progresses from outside to inside, thick vein development and radius are noticed.
(ii) Anisotropy begins during the unstable period of contraction mode.
(iii) The vascular formation does not form until the slime mould contraction mode is no longer regulated by time or place.

The structure of venous and contraction phase bonding stays constant when cells develop naturally. The thickness of the vein is determined in [137] utilizing the Physarum solver and reverse cytoplasm flow.

The increase in cytoplasm suggests an increase in vein diameter. The vein contracts when the flow of cytoplasm decreases, resulting in a drop in the diameter. Slime mould grows stronger in areas where there is more food, ensuring that nutrients are captured with the greatest care. According to the most recent research, slime mould has the ability to forage based on optimization assumption [138]. Slime mould has the capacity to pick higher-concentration nutrition based on food availability and environmental changes. Slime mould, on the other hand, slows things down by leaving the area before foraging. As a result, slime moulds make swift decisions when it comes to protecting the environment [139]. When slime moulds make quick judgements, they take very little time to reach the new region with rich feeding centers, according to rigorous monitoring. When slime moulds make quick judgements, they take very little time to reach the new region with rich feeding centers, according to rigorous monitoring. As a result, while choosing a food source, the slime mould must strike a balance between speed and accuracy. Experiments have demonstrated that slime mould has a lesser chance of leaving an area where it obtains high-quality aliment [140]. Slime mould, on the other hand, may use many food sources at once due to its unique biological distinctiveness. This explains why even if slime mould discovers a superior food area, it may isolate a piece of biomass to pertain for both sources of food at the same time when the top quality aliment is discovered [137]. Slime mould also modulates their search patterns energetically dependent on the accessibility of super food. The slime mould adopts a region limited search technique [141] when the quality of a food source is abundant, focusing its search on foodstuff sources that are now available. If the density of available food is found to be less, then slime mould exits the region in search of other food sources [142]. This adaptable search approach can reflect even more when a variety of meal portions are scattered over the region. The physics and features of the slime mould are mathematically elucidated in the sections that follow.

#### Mathematical modeling of slime mould algorithm

Slime mould algorithm is a metaheuristic algorithm designed on the manners of foraging slime moulds. The slime mould utilizes oscillations biologically to change the cytoplasmic stream through vein to move towards better foodstuff sources, then environs the food and secretes enzymes to collapse it. The activity of slime bacteria in obtaining sustenance is represented by the mathematical model. The mathematical modeling of slime mould algorithm and food searching stages are analyzed under.

*Approaching food* Slime mould should be able to find food point, with the stench there in the atmosphere. To explain the contraction process and characterize its behavior mathematically, the following equations are provided:

1 $$ \overrightarrow {{{\text{SM}}(\tau + 1)}} = \overrightarrow {{{\text{SM}}_{b} (\tau )}} + \overrightarrow {vb} \times (\overrightarrow {W} \times \overrightarrow {{{\text{SM}}_{A} (\tau )}} - \overrightarrow {{{\text{SM}}_{B} (\tau )}} ),x > p $$

2 $$ \overrightarrow {{{\text{SM}}(\tau + 1)}} = \overrightarrow {vc} \times \overrightarrow {{{\text{SM}}(\tau )}} ,x \ge p. $$

Here, $\overrightarrow {vc}$ and $\overrightarrow {vb}$ are two parameter variables, among them $\overrightarrow {vc}$ drop from one to zero and $\overrightarrow {vb}$ lies within the limits $[ - c,c]$. $\tau$ indicates the present repetition. $\overrightarrow {{{\text{SM}}_{b} (\tau )}}$ pinpoints individual location of each element in that region where the stench is utmost, $\overrightarrow {{{\text{SM}}(\tau )}}$ is individual position of slime mould, $\overrightarrow {{{\text{SM}}_{A} }}$ and $\overrightarrow {{{\text{SM}}_{B} }}$ are two independent singles chosen accidentally from group and weight of slime mould is $\overrightarrow {W}$.

The maximum limit $p$ is described in the following equation:

3 $$ p = \tanh \left| {Y(t) - {\text{BF}}} \right|, $$

where $t$ progressively tends to 1, 2… *n*, $Y(t)$ is given as slime mould’s fitness $\overrightarrow {{{\text{SM}}(\tau )}}$, among all iterations BF presents the best fitness. $\overrightarrow {vb}$ is expressed in the following equation as

4 $$ \overrightarrow {vb} = [ - c,c] $$

5 $$ c = \arctan h\left[ { - \left( {\frac{\tau }{{\max_{\tau } }}} \right) + 1} \right]. $$

The expression for $\overrightarrow {W}$ is given as

6 $$ \overrightarrow {{W[{\text{Stench}}\;{\text{Index}}(\tau )]}} = \left\{ \begin{gathered} \hfill 1 + x\log \left( {\frac{{{\text{PF}} - Y(t)}}{{{\text{PF}} - {\text{lF}}}} + 1} \right) \\ \hfill 1 - x\log \left( {\frac{{{\text{PF}} - Y(t)}}{{{\text{PF}} - {\text{lF}}}} + 1} \right) \\ \end{gathered} \right. $$

7 $$ {\text{Stechn}}\;{\text{Index}} = {\text{sort}}(Y). $$

**Fig. 3:**
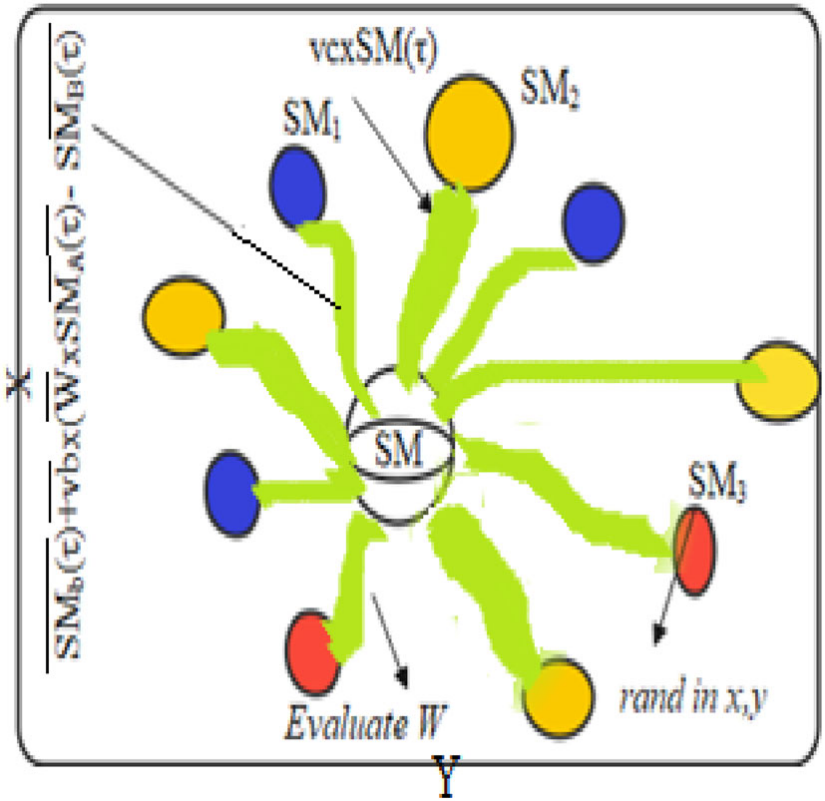
View in two dimensions of a probable position

**Fig. 4:**
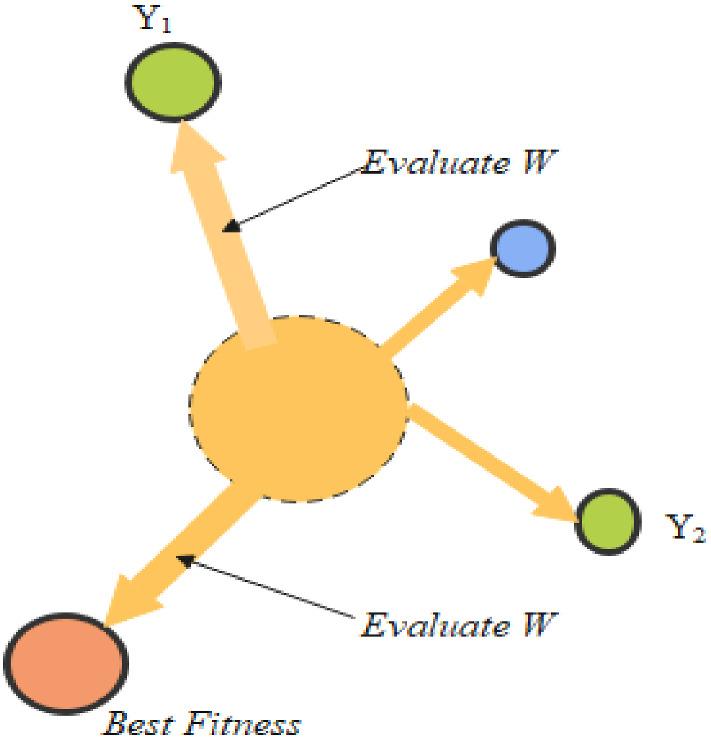
Fitness evaluation

Here, $Y(t)$ in fact rated first half section of the population, $x$ implies its arbitrary number at a period of [0,1], PF indicates optimum fitness attained in the present repetitive procedure, lF symbolizes a low fitness value achieved in the repetitive procedure, stench index reflects the sequence of categorized attributes of fitness. Figures 3 and 4 depict the outcomes of Eqs. (1) and (2) and the probable locations of slime mould in 2D and 3D views. The location of independent $\overrightarrow {{{\text{SM}}(\tau )}}$ may be modified to the finest position $\overrightarrow {{{\text{SM}}_{B} }}$ presently resulted, and altering of $\overrightarrow {W}$, $\overrightarrow {vc}$, and $\overrightarrow {vb}$ will correct the location of the target.

*Wrapping food* This portion technically mimics how the contraction mode of slime mould and venous tissue configuration looks. When the vein gains maximum foodstuff absorption, the healthier the generated wave, the more rapidly the cytoplasm travels in addition to the thicker the vein becomes is shown in Eq. (6). The benefits and fault analysis among the slime mould vein thickness and the food concentration examination was numerically calculated. The $x$ in Eq. (6) represents uncertainty in the reproduction of venous contraction. The rate of change of numerical value is minimized by Log, such that the frequency value of contraction will not update too far. The conditions affect slime mould to improve their styles as per the quality food availability. The weight puts on in the area of high food concentration; if there is a reduction in food concentration, the weight in that area diminishes, thus searching additional sites. Figure 4 portraits fitness evaluation procedure of a slime mould.

The new location of the slime mould is presented mathematically as

8.1 $$ \overrightarrow {{{\text{SM}}^{I} }} = {\text{rand}} \times (U_{ub} - U_{lb} ) + U_{lb} ,{\text{rand}} < Z $$

8.2 $$ \overrightarrow {{{\text{SM}}^{I} }} = \overrightarrow {{{\text{SM}}_{b} (\tau )}} + \overrightarrow {vb} \times (\overrightarrow {W} \times \overrightarrow {{{\text{SM}}_{A} (\tau )}} - \overrightarrow {{{\text{SM}}_{B} (\tau )}} ),x > p $$

8.3 $$ \overrightarrow {{{\text{SM}}^{I} }} = \overrightarrow {vc} \times \overrightarrow {{{\text{SM}}(\tau )}} ,x \ge p. $$

The superior and lesser restrictions of search ranges are specified as $U_{ub}$, $U_{lb}$, and ${\text{rand}}$, and $x$ pinpoints the arbitrary value in the period [0,1].

*Food grabble* Slime mould clearly relies on its own propagation (circulation) wave produced by the phase of oscillations biologically to vary the cytoplasm stream passing through veins, and they seem to absorb food in a better way. To imitate in slime mould the changes of venous width, $\overrightarrow {W}$, $\overrightarrow {vc}$, and $\overrightarrow {vb}$ are proposed to recognize the varieties. The weight $\overrightarrow {W}$ of slime mould indicates its frequency oscillation to creep to different areas of food located, that too slime mould easily reaches the food when the food is abundant and reaches very slowly when the food is very less. This quality in slime mould makes it to find an optimal food source. The parameter $\overrightarrow {vb}$ value varies between $[ - c,c]$ randomly and increases to reach zero when iterations escalate.

The parameter $\overrightarrow {vc}$ value lies in the interval [− 1, 1] and gradually drop to zero when iterations shoot up. To search for the best place for abundant food, slime mould make efforts to explore rich quality food, this gives better solution and keeps moving to find optimum solution.

### Simulated annealing algorithm

The process of simulated annealing (SA) is a probabilistic approach for finding global optimal solution for a function. This metaheuristic-based algorithm in a vast search space nears global optimal value for a given optimization problem. When the search space is discrete, it is usually utilized. Simulated annealing may be superior to procedures like gradient descent or branch and bound for issues where achieving globally optimum solution is also essential than obtaining a local optimum accurately in a limited period. The term annealing derives from the metallurgical procedure of heating and cooling a matter to enhance crystal size and remove defects. Scott Kirkpatrick was the first to create this approach, which was given the term simulated annealing algorithm. When accurate approaches fail, simulated annealing can be used to tackle exceedingly complex computational optimization problems; while it only offers an approximate solution to the global minimum, it may suffice in many practical cases.

The computation first generates arbitrary arrangement vectors, known as the beginning arrangement, and then a preset neighborhood arrangement generates another neighbor arrangement, which is also evaluated using a target task. If the neighbor is more powerful than the initial arrangement, the improved advancement is always recognized; however, a more terrible neighbor is always acknowledged with a specific probability controlled by the Boltzmann likelihood using condition in the following equation:

9 $$ T = \alpha * T,\;{\text{here}}\;\alpha = 0.93, $$

where $\alpha$ = 0.93 is the distinction between the wellness of the created neighbor arrangement and best arrangement and diminishing as indicated by cooling plan and *T* is the temperature. The steps for simulated annealing are given as follows.

### Slime mould-simulated annealing algorithm

In the suggested hybridized slime mould-simulated annealing algorithm, the position vector resulting from Eqs. (8.1, 8.2, 8.3) is updated by the suggested hybrid hSMA-SA technique, and the latest location vector is adept on slime mould to compute the food sources in three stages: looming food, bind food, food grabble. The reason behind the merge of the simulated annealing technique with slime mould algorithm is to develop the actual slime mould’s exploitation phase, which has excellent global hunt ability but poor local hunt capacity. Meanwhile, it is also necessary to memorize that the simulated annealing algorithm has a strong local hunt aptitude but a weak global hunt capacity. To amalgamate SMA and SA, a heuristic strategy is chosen in which the simulated annealing method is engaged immediately successive to slime mould. Subsequently, it is noticed that the local hunt ability of traditional slime mould enhanced in the phase of exploitation in obtaining better results when blended with simulated annealing. In order to attain improvement in the suggested algorithm, the principal temperature is treated as $2 * \left| N \right|$. Here, $\left| N \right|$ nominates no. of attributes for the individual dataset.

The cooling itinerary for the simulated annealing method is determined using the equation presented as follows:

10 $$ T = \alpha * T. $$

Depending on the sensitiveness in fluctuations of system energies, the temperature (*T*) controls the system’s state (*S*) evolution. ‘*S*’ the evolution is receptive to boorish energy variations if the value of ‘*T*’ is huge and if the value of ‘*T*’ is tiny, then ‘*S*’ the evolution is receptive for better energy variations. Primarily, 1.0 is the value of temperature in the beginning and it gets multiplied by a constant ‘*α*’ to minimize temperature (*T*) at the closing stages of iteration. The limit range of a constant ‘*α*’ is between 0.8 and 0.99. In this study, the constant ‘*α*’ value is picked as 0.93.

**Fig. 5:**
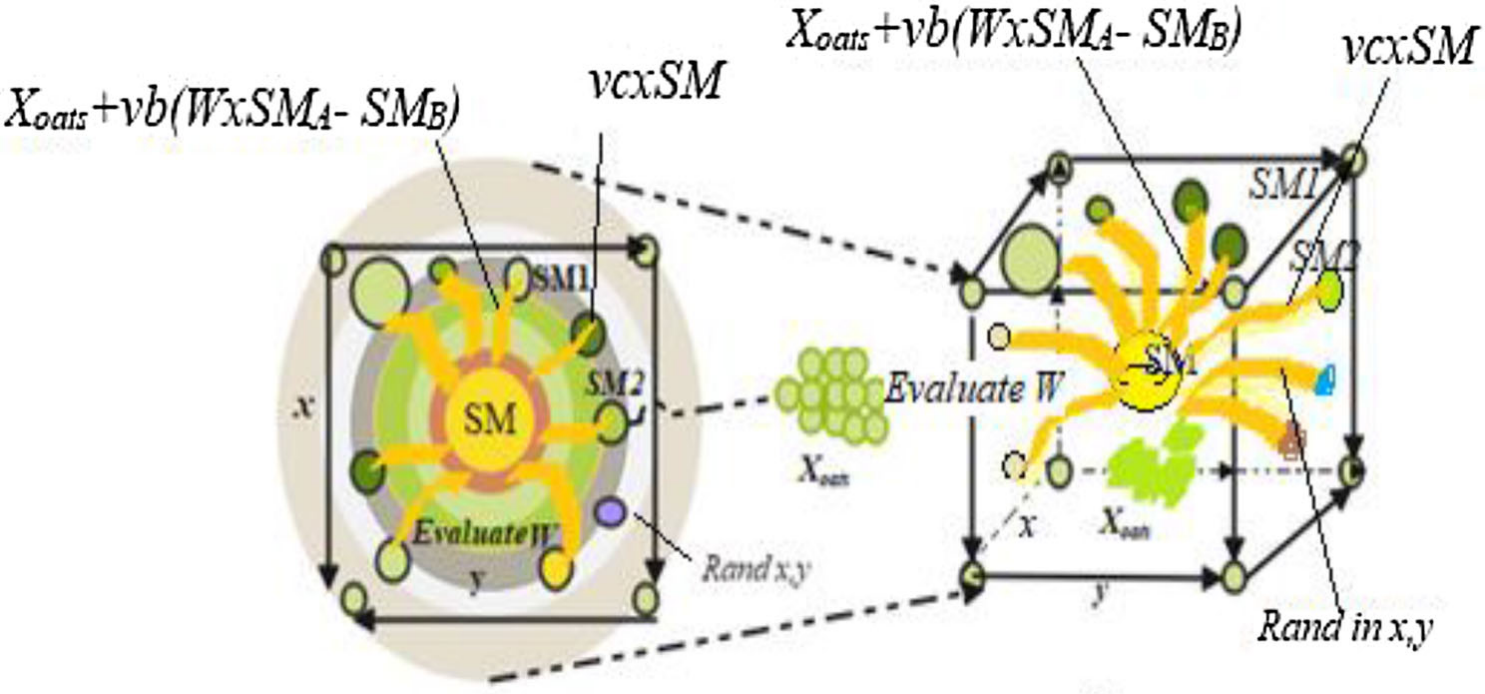
Probable positions in 2D and 3 D

**Fig. 6:**
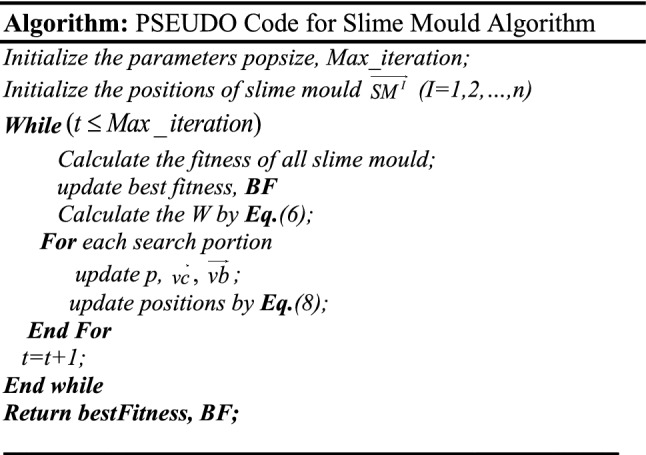
SMA PSEUDO code

**Fig. 7:**
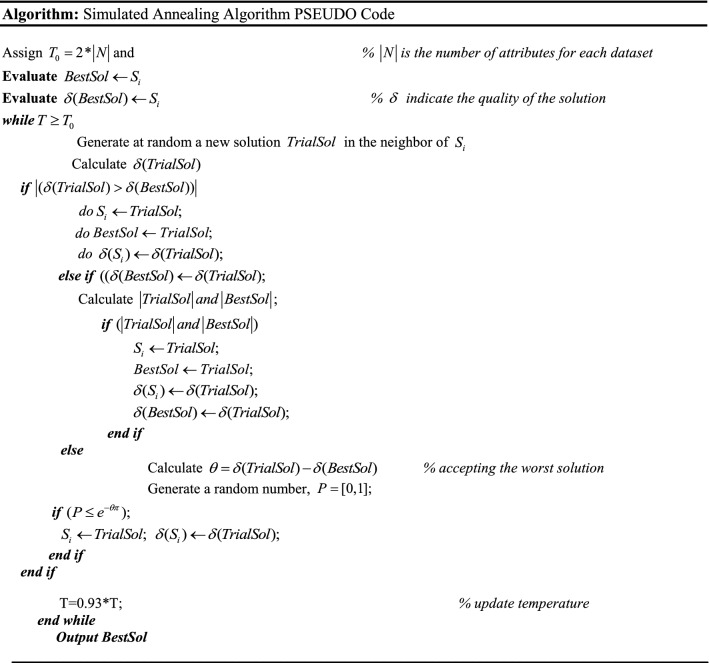
Simulated annealing algorithm PSEUDO code

**Fig. 8:**
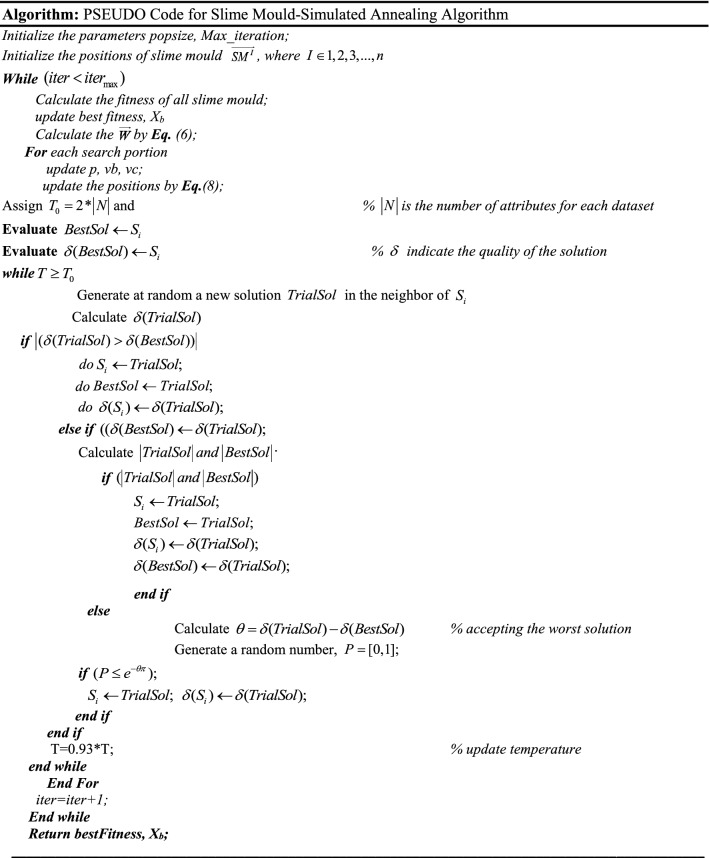
PSEUDO code for hSMA-SA algorithm

**Fig. 9:**
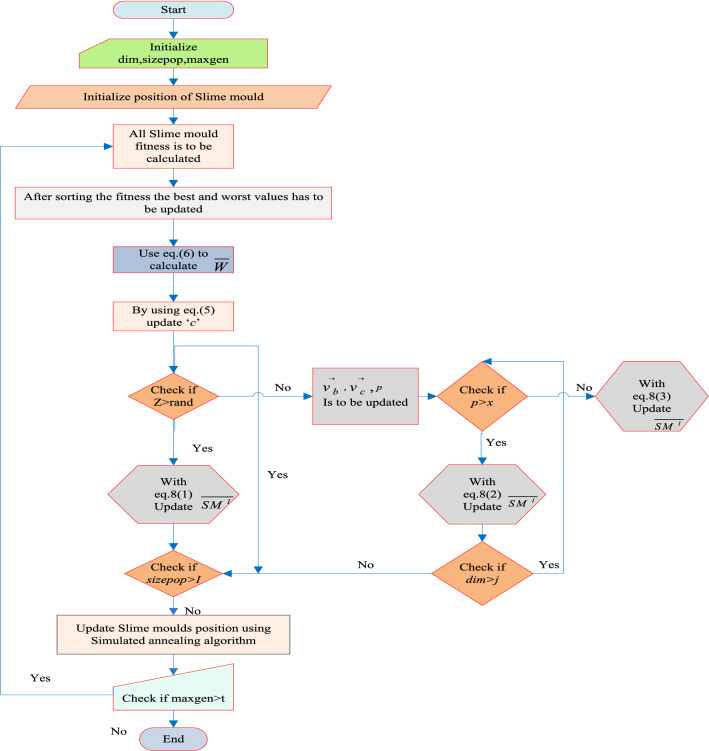
Flow chart for hSMA-SA algorithm

In Fig. 5, a slime mould of (*X*, *Y*) adjusts its location according to latest achieved location vectors and remains in contact with them as described in 2D and 3D views and also defines the location of the food (X*, Y*) as well as develops the search region in improvised means. Improved positions are gained by estimating the vectors $\vec{a}$ and $\vec{c}$. The hSMA-SA explorative phase is same as traditional SMA. Vectors $\vec{a}$ and $\vec{c}$ are utilized to search globally in technical model divergence (Fig. 6). Slime mould gets expanded in the environment in search of food when vector $\vec{a}$ is greater than 1 (Fig. 7). The developed hSMA-SA algorithm PSEUDO code is showcased in Figs. 8, and 9 displays the flow chart.

## Standard benchmark functions

The suggested hSMA-SA optimization strategy is put to the test using a cluster of distinct benchmark functions [143]. Standard benchmarks are divided into three categories: unimodal (UM), multimodal (MM), and fixed dimensions (FD). For these benchmark functions, the size, range limit, and optimal value, are determined based on objective fitness (*f*_min_). Tables 2, 3, and 4 show the numerical formulations for UM, MM, and FD, respectively, and the findings are given in the outcomes and discussion section. The performance of typical benchmark functions is evaluated using 30 trial runs. The details of parameter setup for the proposed method are shown in Table 5.

**Table 2:** Unimodal standard benchmark functions

Functions	Dimensions	Range	*f*_min_
$F_{1} (U) = \sum\nolimits_{m = 1}^{z} {U_{m}^{2} }$	30	[− 100, 100]	0
$F_{2} (U) = \sum\nolimits_{m = 1}^{z} {\left| {U_{m} } \right|} + \mathop \prod \nolimits_{m = 1}^{z} \left| {U_{m} } \right|$	30	[− 10,10]	0
$F_{3} (U) = \sum\nolimits_{m = 1}^{z} {\left( {\sum\nolimits_{n - 1}^{m} {U_{n} } } \right)^{2} }$	30	[− 100, 100]	0
$F_{4} (U) = \max_{m} \{ \left| {U_{m} } \right|,1 \le m \le z\}$	30	[− 100, 100]	0
$F_{5} (U) = \sum\nolimits_{m = 1}^{z - 1} {[100(U_{m + 1} } - U_{m}^{2} )^{2} + (U_{m} - 1)^{2} ]$	30	[− 38, 38]	0
$F_{6} (U) = \sum\nolimits_{m = 1}^{z} {([U_{m} } + 0.5])^{2}$	30	[− 100, 100]	0
$F_{7} (U) = \sum\nolimits_{m = 1}^{z} {mU_{m}^{4} } + {\text{random}}[0,1]$	30	[− 1.28, 1.28]	0

**Table 3:** Multimodal standard benchmark functions

Multimodal bench mark functions	Dim	Range	$f_{\min }$
$F_{8} (U) = \sum\nolimits_{m = 1}^{z} { - U_{m} } \sin (\sqrt {\left| {U_{m} } \right|} )$	30	[− 500, 500]	− 418.98295
$F_{9} (U) = \sum\nolimits_{m = 1}^{z} {[U_{m}^{2} - 10\cos (2\pi U_{m} ) + 10]}$	30	[− 5.12, 5.12]	0
$F_{10} (U) = - 20\exp \left( { - 0.2\sqrt {\left( {\frac{1}{z}\sum\nolimits_{m = 1}^{z} {U_{m}^{2} } } \right)} } \right) - \exp \left( {\frac{1}{z}\sum\nolimits_{m = 1}^{z} {\cos (2\pi U_{m} } } \right) + 20 + d$	30	[− 32, 32]	0
$F_{11} (U) = 1 + \sum\nolimits_{m = 1}^{z} {\frac{{U_{m}^{2} }}{4000}} - \prod_{m - 1}^{z} \cos \frac{{U_{m} }}{\sqrt m }$	30	[− 600, 600]	0
$$\begin{aligned} F_{12} (U) & = \frac{\pi }{z}\left\{ {10\sin (\pi \tau_{1} ) + \sum\nolimits_{m = 1}^{z - 1} {(\tau_{m} - 1)^{2} [1 + 10\sin^{2} (\pi \tau_{m + 1} )] + (\tau_{z} - 1)^{2} } } \right\} \\ & \quad + \sum\nolimits_{m = 1}^{z} {g(U_{m} ,10,100,4)} \\ \end{aligned}$$ where $\tau_{m} = 1 + \frac{{U_{m} + 1}}{4}$ $$g(U_{m} ,b,x,i) = \left\{ {0\begin{array}{*{20}c} {x(U_{m} - b)^{i} U_{m} > b} \\ { - b < U_{m} < b} \\ {x( - U_{m} - b)^{i} U_{m} < - b} \\ \end{array} } \right\}$$	30	[− 50, 50]	0
$F_{13} (U) = 0.1\left\{ {\sin^{2} (3\pi U_{m} ) + \sum\nolimits_{m = 1}^{z} {(U_{m} - 1)^{2} [1 + \sin^{2} (3\pi U_{m} + 1)] + (x_{z} - 1)^{2} [1 + \sin^{2} ]} } \right\}$	30	[− 50, 50]	0

**Table 4:** Fixed-dimension benchmark functions

Fixed-dimension (FD) benchmark functions	Dimension	Range	$f_{\min }$
$F_{14} (U) = \left[ {\frac{1}{500} + \sum\nolimits_{n = 1}^{2} 5 \frac{1}{{n + \sum\nolimits_{m = 1}^{z} {(U_{m} - b_{mn} )6} }}} \right]^{ - 1}$	2	[− 65.536, 65.536]	1
$F_{15} (U) = \sum\nolimits_{m = 1}^{11} {\left[ {b_{m} - \frac{{U_{1} (a_{m}^{2} + a_{m} \eta_{2} )}}{{a_{m}^{2} + a_{m} \eta_{3} + \eta_{4} }}} \right]}^{2}$	4	[− 5, 5]	0.00030
$F_{16} (U) = 4U_{1}^{2} - 2.1U_{1}^{4} + \frac{1}{3}U_{1}^{6} + U_{1} U_{2} - 4U_{2}^{2} + 4U_{2}^{4}$	2	[− 5, 5]	− 1.0316
$F_{17} (U) = \left( {U_{2} - \frac{5.1}{{4\pi 2}}U_{1}^{2} + \frac{5}{\pi }U_{1} - 6} \right)^{2} + 10\left( {1 - \frac{1}{8\pi }} \right)\cos U_{1} + 10$	2	[− 5, 5]	0.398
$$\begin{gathered} F_{18} (U) = [1 + (U_{1} + U_{2} + 1)^{2} (19 - 14U_{1} + 3U_{1}^{2} - 14U_{2} + 6U_{1} U_{2} + 3U_{2}^{2} )] \hfill \\ x[30 + (2U_{1} - 3U_{2} )^{2} x(18 - 32U_{1} + 12U_{1}^{2} + 48U_{2} - 36U_{1} U_{2} + 27U_{2}^{2} )] \hfill \\ \end{gathered}$$	2	[− 2,2]	3
$F_{19} (U) = - \sum\nolimits_{m = 1}^{4} {d_{m} \exp ( - \sum\nolimits_{n = 1}^{3} {U_{mn} (U_{m} - q_{mn} )^{2} } } )$	3	[1, 3]	− 3.32
$F_{20} (U) = - \sum\nolimits_{m = 1}^{4} {d_{m} } \exp ( - \sum\nolimits_{n = 1}^{6} {U_{mn} (U_{m} } - q_{mn} )^{2} )$	6	[0, 1]	− 3.32
$F_{21} (U) = - \sum\nolimits_{m = 1}^{5} {[(U - b_{m} )(U - b_{m} )^{T} + d_{m} } ]^{ - 1}$	4	[0,10]	− 10.1532
$F_{22} (U) = - \sum\nolimits_{m = 1}^{7} {[(U - b_{m} )(U - b_{m} )^{T} } + d_{m} ]^{ - 1}$	4	[0, 10]	− 10.4028
$F_{23} (U) = - \sum\nolimits_{m = 1}^{7} {[(U - b_{m} )(U - b_{m} )^{T} } + d_{m} ]^{ - 1}$	4	[0, 10]	− 10.5363

**Table 5:** Parameter constraints for the suggested technique

Parameter setting	hSMA-SA
Search agents	30
Count of iterations for benchmark problems (unimodal, multimodal and fixed dimension)	500
Count of iterations for engineering optimal designs	500
Count of trial runs for each function and engineering optimal designs	30

Thirty search agents are used to go through the entire research, with a maximum of 500 iterations. The proposed hSMA-SA was evaluated using the MATLAB R2016a program using a laptop of Intel corei3 processor with an 8 GB RAM and 7th generation CPU.

According to the results of the comparative study, the suggested heuristic technique significantly boosts the rate of convergence as well as develops its capacity to quickly run away from local area stagnation.

## Results and analysis

The offered slime mould-simulated annealing method is assessed on three primary modules of customary benchmark functions in this study effort to validate the success rate of the suggested hSMA-SA technique. The exploitation and convergence rate of hSMA-SA are assessed using unimodal benchmark functions with a single optimal solution. As the name indicates, multimodal replicates several perfect solutions, as a result, these can be used to check for exploration and prevent finding a local optimal solution. The distinction among multimodal and fixed-dimension benchmark functions determines the design variables. These design variables will be saved in fixed-dimension benchmark functions, which will uphold a graphic representation of preceding search space data to compare with multimodal functions.

For detailed study, a documentation of the outcomes for the launched hSMA-SA technique was supplied; in the table form indicating statistical outputs, time of computation, and evaluation of the technique by executing with 500 iterations and 30 runs.

### Evaluation of unimodal functions (exploitation)

The search progress for the finest place stands upon the potential of search agents to arrive nearer to source. At the time of the search procedure, there is a chance for various agents to get ensnare far or nearby in view of the phases exploration and exploitation. Exploration falls below global search whereas exploitation refers to local search. The results of unimodal functions have a statistical analysis in selected points such as search record, convergence behavior, average fitness of population. The search record in the trail runs graph shows the locations of slime mould. The graph of convergence explains the variation in the position of slime mould during optimization procedure. The average fitness of the population describes the variations in the average population during whole optimization procedure. This better convergence certifies the effectiveness of the suggested algorithm. The low *p* value shown in Table 9, which was acquired using the statistical Wilcoxon rank sum test and *t* test to examine the proposed algorithm’s detailed behavior, indicates that the produced algorithm has better convergence and is more effective. At a 95% level of significance, the *h* value further supports the null hypothesis. The suggested algorithm’s parametric test demonstrates that the null hypothesis is rejected at the alpha significance level. If *h* = 1, the null hypothesis has been rejected at the alpha significance level. If *h* = 0, the null hypothesis was not successfully rejected at the alpha significance level.

**Fig. 10:**
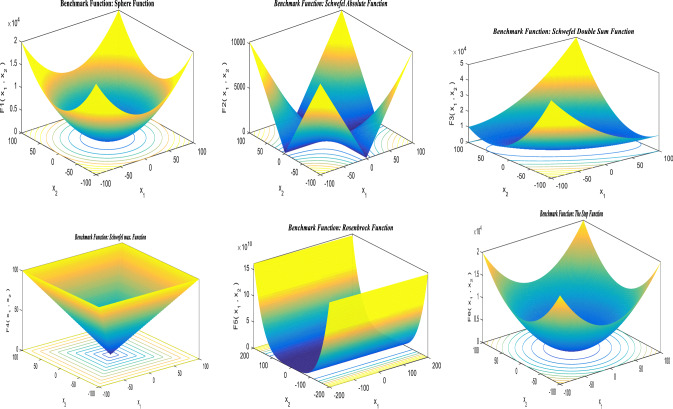
3D view of unimodal functions

**Fig. 11:**
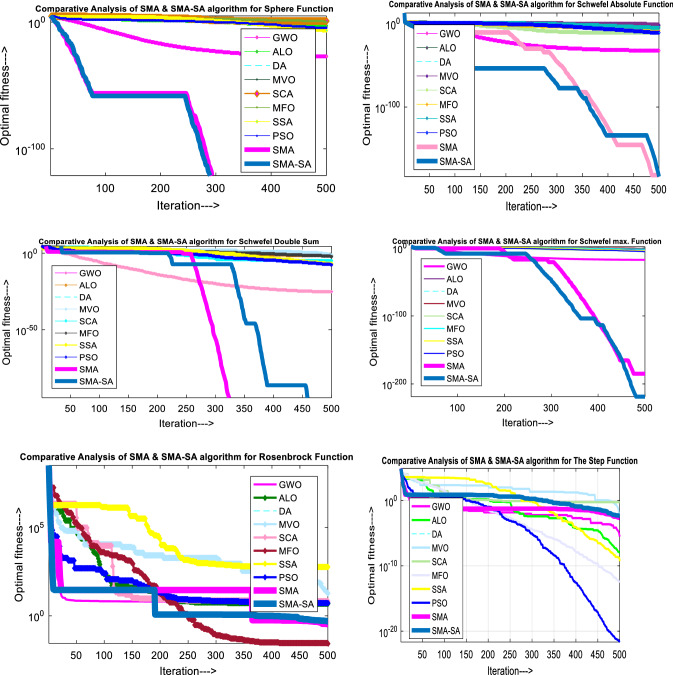
Convergence curve of hSMA-SA with known algorithms for F1–F6 functions

**Fig. 12:**
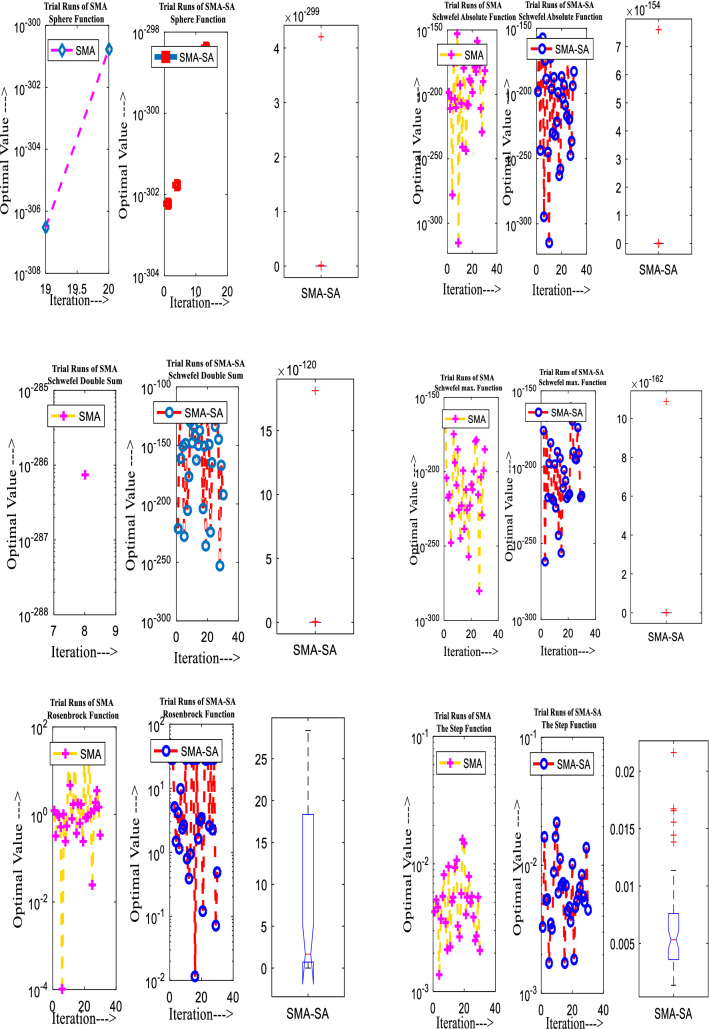
Trial runs of SMA and hSMA-SA for F1–F6 functions

Figure 10 showcases the characteristic curves of unimodal benchmark functions and Fig. 11 shows a comparison of hSMA-SA with other different algorithms. It is observed from the curves of convergence of proposed algorithm converges to optimum very soon. To ensure the aptness of the launched technique, every test function is assumed with SMA and SA. The statistical outputs in Table 6 exhibit the unimodal functions in view of mean, standard deviation, best fitness value, worst fitness, median, *p* value and *t* value. There are a few areas of global optima and a few areas get jammed in local optima in search region. The global search procedure finds the exploration phase while the local search procedure explores the exploitation. The appraisal of any technique is inspected by its capability in attaining maxima or minima within a little time of computation. Table 7 displays the time of computation in view of best, average as well as worst time. Table 8 displays the evaluation of hSMA-SA technique with other already available methods such as LSA [144], (SCA) [102], BRO [145], DA [146], OEGWO [147], MFO [34], PSA [74], HHO-PS [50], (SSA) [148], SHO [46], GWO [149], HHO [78], MVO [24], ECSA [150], PSO [151], TSO [109], ALO [152], and LF-SMA [8] considering standard deviation and average value. There is a variation in benchmark functions in view of characteristics. All these functions differ in their search abilities in the zones of exploration and exploitation. In this context, test judgment for six unimodal benchmark functions is examined. The test results for each function are reported in terms of average and standard deviation after 30 trial runs and 500 iterations. To examine the influence of SA on the solutions of hSMA-SA the scalability measurement is conceded. Table 8 displaying the statistical result announces a significant gap between hSMA-SA and other techniques. It is clear from Table 8 that by injecting SA technique, the SMA gained strength to enhance exploration and exploitation phases. The results of hSMA-SA when compared with SCA, ALO, PSA, SSA, MVO, BRO, PSO, MFO, DA, and GWO show noteworthy feat in handling with F3, F5, and F6 test functions in terms of standard deviation and average value. According to Fig. 11 convergence curves, it is noticed that with enhanced efficacy, the optimality results shoot up. The former approaches shown converge early. Moreover, to prove the success of the introduced method, every benchmark function’s independent trial runs are shown in Fig. 12. By comparison, it is proved that the SA algorithm promotes to investigate the local search phase with high intensity.

**Table 6:** Test note for unimodal functions using hSMA-SA technique

Function	Mean	Standard deviation	Best fitness value	Worst fitness value	Median	Wilcoxon rank sum test	*t* test	
						*p* value	*p* value	*h* value
Sphere function (F1)	1.4E−300	0	0	4.2059E−299	0	0.125	0	1
Schwefel absolute function (F2)	1.9E−159	1.0209E−158	0	5.5926E−158	4.6226E−200	1.7344E−06	0.32386916	0
Schwefel double sum function (F3)	6.1E−121	3.309E−120	0	1.8126E−119	2.5788E−151	2.56308E−06	0.324043719	0
Schwefel max. function (F4)	3.6E−163	2.2228E−162	1.0007E−262	1.088E−161	4.4998E−199	1.7344E−06	0.378435872	0
Rosenbrock function (F5)	11.77822	12.89147042	0.011463167	28.35510393	3.286513444	1.7344E−06	2.50694E−05	1
The step function (F6)	0.007059	0.004749705	0.001719961	0.021628358	0.005572663	1.73E−06	5.63E−09	1

**Table 7:** Time for execution for unimodal functions using hSMA-SA technique

Function	Best time	Average time	Worst time
Sphere function (F1)	190.3906	219.1385417	297.39063
Schwefel absolute function (F2)	122.7188	133.9958333	155.51563
Schwefel double sum function (F3)	137.2188	144.546875	162.70313
Schwefel max. function (F4)	111.875	176.4671875	307.20313
Rosenbrock function (F5)	90.25	112.4067708	150.95313
The step function (F6)	195.25	212.9197917	327.8125

**Table 8:** Evaluation for unimodal problems

Algorithm	Parameters	Unimodal Benchmark functions					
		sphere function (F1)	Schwefel absolute function (F2)	Schwefel double sum function (F3)	Schwefel max. function (F4)	Rosenbrock function (F5)	The step function (F6)
Lightning search algorithm (LSA) [144]	Avg	4.81067E−08	3.340000000	0.024079674	0.036806544	43.24080402	1.493275733
	S. deviation	3.40126E−07	2.086007800	0.005726198	0.156233023	29.92194448	1.302827039
Dragonfly algorithm (DA) [146]	Avg	2.850E−19	1.490E−06	1.290E−07	9.88E−04	7.6	4.170E−17
	S. deviation	7.160E−19	3.760E−06	2.100E−07	2.78E−03	6.79	1.320E−16
Battle Royale optimization algorithm (BRO) [145]	Avg	3.0353E−09	0.000046	54.865255	0.518757	99.936848	2.8731E−08
	S. deviation	4.1348E−09	0.000024	16.117329	0.403657	82.862958	1.8423E−08
Multi-verse optimizer (MVO) [24]	Avg	2.08583	15.9247	453.200	3.12301	1272.13	2.29495
	S. deviation	0.64865	44.7459	177.0973	1.58291	1479.47	0.63081
Opposition-based enhanced grey wolf optimization algorithm (OEGWO) [147]	Avg	2.49 × 10^–34^	4.90 × 10^–25^	1.01 × 10^–1^	1.90 × 10^–5^	2.72 × 10^1^	1.40 × 10^00^
	S. deviation	7.90 × 10^–34^	6.63 × 10^–25^	3.21 × 10^–1^	2.43 × 10^–5^	7.85 × 10^1^	4.91 × 10^–1^
Particle swarm optimization (PSO) [151]	Avg	1.3E−04	0.04214	7.01256E+01	1.08648	96.7183	0.00010
	S. deviation	0.0002.0E−04	0.04542	2.1192E+01	3.1703E+01	6.01155E+01	8.28E−05
Photon search algorithm (PSA) [74]	Avg	15.3222	2.2314	3978.0837	1.1947	332.6410	19.8667
	S. deviation	27.3389	1.5088	3718.9156	1.0316	705.1589	33.4589
Sine–cosine algorithm (SCA) [102]	Avg	0.000	0.000	0.0371	0.0965	0.0005	0.0002
	S. deviation	0.000	0.0001	0.1372	0.5823	0.0017	0.0001
Hybrid Harris hawks optimizer–pattern search algorithm (hHHO-PS) [50]	Avg	9.2 × 10^–017^	8.31E	5.03 × 10^–20^	6.20 × 10^–54^	2.18 × 10^–9^	3.95 × 10^–14^
	S. deviation	5E−106	4.46 × 10^–53^	1.12 × 10^–19^	1.75 × 10^–53^	6.38 × 10^–10^	3.61 × 10^–14^
Ant lion optimizer (ALO) [152]	Avg	2.59E−10	1.84E−06	6.07E−10	1.36E−08	0.3467724	2.56E−10
	S. deviation	1.65E−10	6.58E−07	6.34E−10	1.81E−09	0.10958	1.09E−10
Spotted hyena optimizer (SHO) [46]	Avg	0	0	0	7.78E−12	8.59E+00	2.46E−01
	S. deviation	0	0	0	8.96E−12	5.53E−01	1.78E−01
Moth flame optimizer (MFO) [34]	Avg	0.00011	0.00063	696.730	70.6864	139.1487	0.000113
	S. deviation	0.00015	0.00087	188.527	5.27505	120.2607	9.87E−05
Harris hawks optimizer (HHO) [78]	Avg	1.06 × 10^–90^	6.92 × 10^–51^	1.25 × 10^–80^	4.46 × 10^–48^	0.015002	0.000115
	S. deviation	5.82 × 10^–90^	2.47 × 10^–50^	6.63 × 10^–80^	1.70 × 10^–47^	0.023473	0.000154
Grey wolf optimizer (GWO) [149]	Avg	6.590E−29	7.180E−18	3.20E−−07	5.610E−08	26.8125	0.81657
	S. deviation	6.3400E−07	0.02901	7.9.1495E+01	1.31508	69.9049	0.00012
Enhanced crow search algorithm (ECSA) [150]	Avg	7.4323E−119	5.22838E−59	3.194E−102	3.04708E−52	7.996457081	0.400119079
	S. deviation	4.2695E−118	2.86361E−58	1.7494E−101	1.66895E−51	0.661378213	0.193939866
Salp swarm algorithm (SSA) [148]	Avg	0.000	0.2272	0.000	0.000	0.000	0.000
	S. deviation	0.000	1.000	0.000	0.6556	0.000	0.000
Transient search optimization (TSO) [109]	Avg	1.18 × 10^–99^	8.44 × 10^–59^	3.45 × 10^41^	1.28E−53	8.10 × 10^–2^	3.35 × 10^–3^
	S. deviation	6.44 × 10^–99^	3.93 × 10^–58^	1.26 × 10^–41^	6.58 × 10^–53^	11	6.82 × 10^–3^
LF-SMA [8]	Avg	1.58E−156	2.74E−171	5.2412	0.0006	5.90E−05	0.0008
	S. deviation	7.53E−156	0	10.229	0.0002	6.38E−05	0.0008
Proposed algorithm hSMA-SA	Avg	1.4E−300	1.9E−159	6.1E−121	3.6E−163	11.77822	0.007059
	S. deviation	**0**	1.0209E−158	3.309E−120	2.2228E−162	12.89147042	0.004749705

**Table 9:** Test results for multimodal functions using hSMA-SA technique

Function	Mean	Standard deviation	Best fitness value	Worst fitness value	Median	Wilcoxon rank sum test	*t* test	
						*p* value	*p* value	*h* value
Schwefel sine function (F8)	− 12,569.02623	0.43623993	− 12,569.48529	− 12,567.9831	− 12,569.15434	1.73E−06	4.22E−131	1
Rastrigin function (F9)	0	0	0	0	0	1	0	1
The Ackley function (F10)	8.88E−16	0	8.88E−16	8.88E−16	8.88E−16	4.32E−08	0	1
Penalized penalty#1 function (F12)	0.012678845	0.012483005	9.69E−05	0.039727727	0.007266933	1.73E−06	5.31E−06	1
Levi N. 13 function (F13)	0.002689783	0.001733379	0.000388677	0.007871366	0.002737656	1.73E−06	2.30E−09	1

### Evaluation of a few multimodal functions (exploration)

**Fig. 13:**
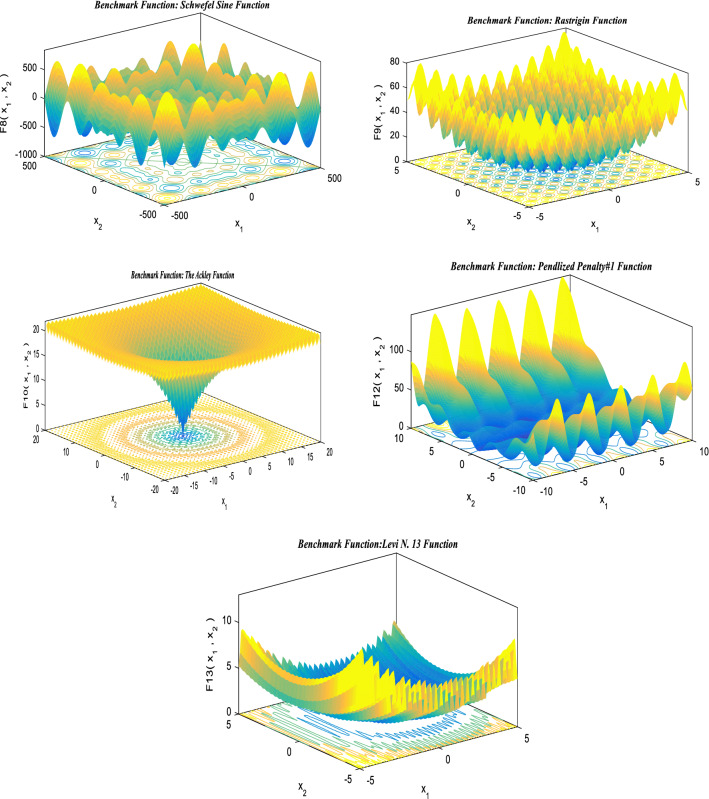
3D view of multimodal standard benchmark functions

**Fig. 14:**
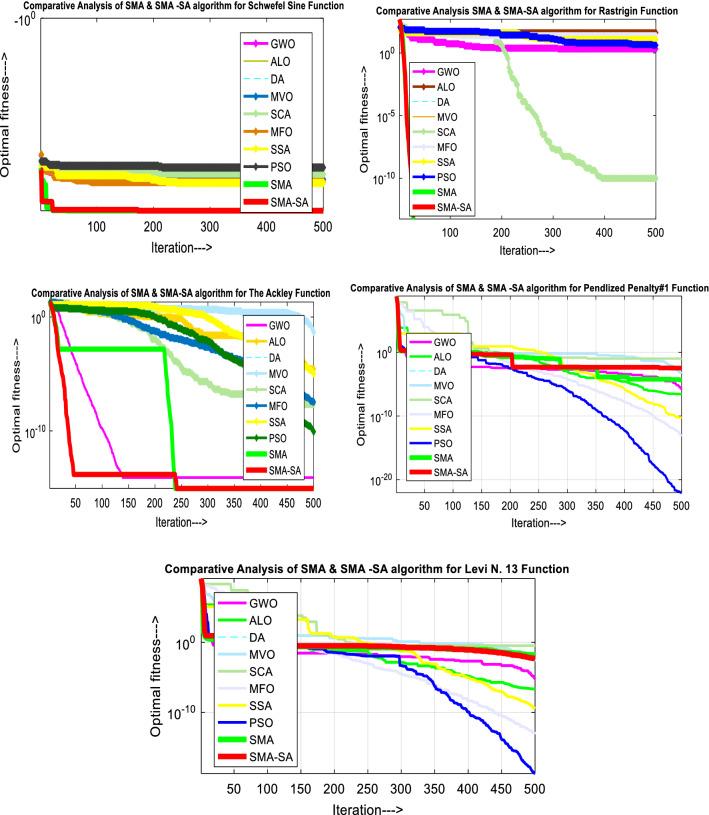
Convergence curve of hSMA-SA with known algorithms for multimodal functions

**Fig. 15:**
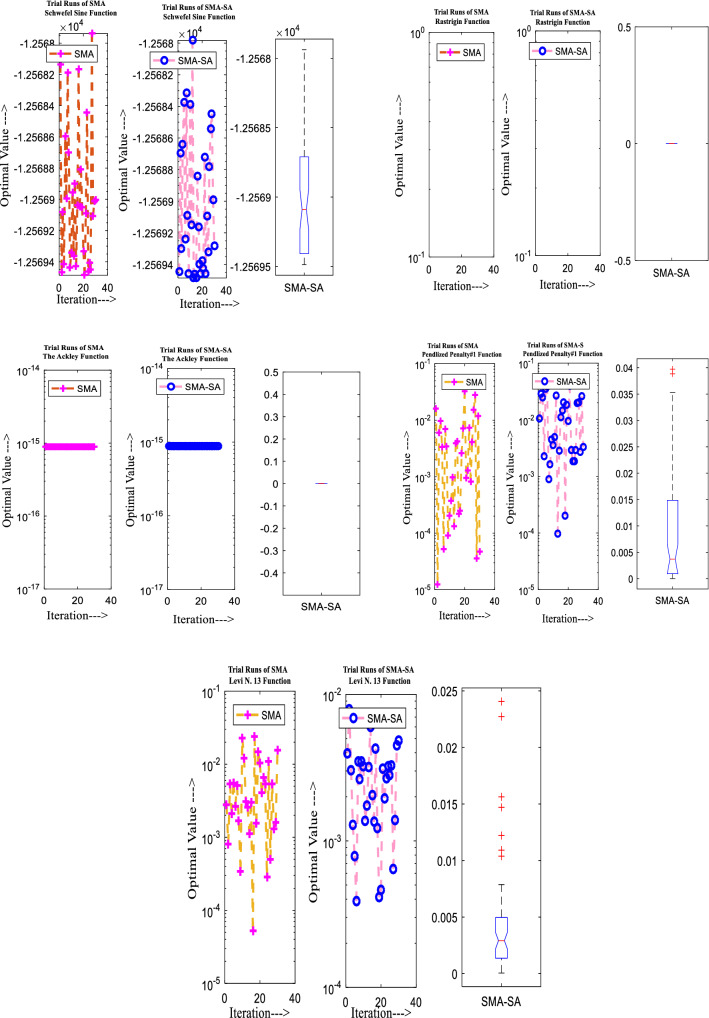
Trial runs of SMA and hSMA-SA for multimodal benchmark functions

Figure 13 showcases the characteristic curves of multimodal benchmark functions and Fig. 14 presents comparison between hSMA-SA and other techniques. In this study, F8, F9, F10, F12, and F13 multimodal benchmark functions are examined considering 30 trial runs with 500 iterations and the results are tabulated in Table 9. The execution time for the simulation process for multimodal are recorded and tabled in Table 10. Table 11 contains the information of compared results of hSMA-SA algorithm with other already available metaheuristic search algorithms such as LSA [144], (SCA) [102], BRO [145], DA [146], OEGWO [147], MFO [34], PSA [74], HHO-PS [50], (SSA) [148], SHO [46], GWO [149], HHO [78], MVO [24], ECSA [150], PSO [151], TSO [109], ALO [152], and LF-SMA [8] considering standard deviation and average value. The results of multimodal functions have a statistical analysis in selected points such as search record, convergence behavior, and average fitness of population. The search record in the trail runs graph shows the positions of slime mould. The graph of convergence explains the variation in the position of slime mould during optimization procedure. The average fitness of the population describes the changes in the average population during whole optimization procedure. This better convergence certifies the effectiveness of the suggested algorithm. The created algorithm has better convergence and is more efficient, according to the low *p* value supplied in Table 9, which was obtained using the statistical Wilcoxon rank sum test and *t* test to check the recommended algorithm’s detailed behavior. The *h* value further validates the null hypothesis at a 95% level of significance. The null hypothesis is rejected at the alpha significance level by the parametric test of the provided method. At the alpha significance level, the null hypothesis has been rejected if *h* = 1. If *h* = 0, the alpha significance threshold did not allow for a valid rejection of the null hypothesis. It is noted from Fig. 14 that the results of F8, F9, F10, F12, and F13 multimodal benchmark functions have improved convergence curves using hSMA-SA, justifying the aptness of the algorithm in verdict solutions for the multimodal functions. Table 11 shows the analysis of statistical data revealing that the optimality of multimodal benchmark functions is slightly gained better results by applying SA algorithm. The average and standard deviation outcomes reveal that hSMA-SA performs better for all selected five test functions than ECSA, HHO-PS, and LSA. It is recorded from the convergence curves of Fig. 14 that hSMA-SA allows optimal convergences for a few test functions. From the comparison of convergence, it is well understood that hSMA-SA converges soon and catches the run as early as it attains the end condition. From the comparative curves of Fig. 14, it is noticeable that the proposed technique acts good in handling F9, F10, and F13 and performs comparatively better than F8 and F12. Moreover, to prove the success of the proposed technique, every benchmark function’s independent trial runs are shown in Fig. 15. The comparison study proved that hSMA-SA algorithm appreciably searches with extra intensity in the local and global search space.

**Table 10:** Time of execution for multimodal functions via hSMA-SA technique

Function	Best time	Average time	Worst time
Schwefel sine function (F8)	659.2031	666.775	686.51563
Rastrigin function (F9)	513.2031	532.9604167	586.29688
The Ackley function (F10)	566.0469	575.2671875	599.53125
Penalized penalty#1 function (F12)	2008.016	2101.541146	2290.125
Levi N. 13 function (F13)	2062.094	2145.145313	2304.7031

**Table 11:** Comparison for multimodal benchmark functions

Algorithm	Parameters	Multimodal benchmark functions				
		Schwefel sine function (F8)	Rastrigin function (F9)	The Ackley function (F10)	Penalized penalty#1 function (F12)	Levi N. 13 function (F13)
Lightning search algorithm (LSA) [144]	Avg	− 8001.3887	62.7618960	1.077446947	2.686199995	0.007241875
	St. deviation	669.159310	14.9153021	0.337979509	0.910802774	0.006753356
Dragonfly algorithm (DA) [146]	Avg	− 2.860E+03	1.600E+01	2.310E−01	3.110E−02	2.200E−03
	St. deviation	3.840E+02	9.480E+00	4.870E−01	9.830E−02	4.630E−03
Battle Royale optimization algorithm (BRO) [145]	Avg	− 7035.2107	48.275350	0.350724	0.369497	0.000004
	St. deviation	712.33269	14.094585	0.688702	0.601450	0.000020
Multi-verse optimizer (MVO) [24]	Avg	− 1.170E+04	1.180E+02	4.070E+00	2.460E+00	2.200E−01
	St. deviation	9.370E+02	3.930E+01	5.500E+00	7.900E−01	9.000E−02
Opposition-based enhanced grey wolf optimization algorithm (OEGWO) [147]	Avg	− 3.36 × 10^3^	8.48 × 10^–1^	9.41 × 10^–15^	9.36 × 10^–02^	1.24E+00
	St. deviation	3.53 × 10^2^	4.65E+00	3.56 × 10^–15^	3.95 × 10^–02^	2.09 × 10^–1^
Particle swarm optimization (PSO) [151]	Avg	− 4.8400E+04	4.670E+01	2.760E−01	6.9200E−04	6.6800E−04
	St. deviation	1.1500E+04	1.160E+01	5.090E−01	2.6300E−03	8.9100E−04
Photon search algorithm (PSA) [74]	Avg	11,648.5512	7.3763	1.6766	0.1716	1.5458
	St. deviation	1230.4314	9.1989	0.9929	0.2706	3.3136
Sine–cosine algorithm (SCA) [102]	Avg	1.000E+00	0.000E+00	3.800E−01	0.000E+00	0.000E+00
	St. deviation	3.600E−03	7.300E−01	1.000E+00	0.000E+00	0.000E+00
Hybrid Harris hawks optimizer–pattern search algorithm (hHHO-PS) [50]	Avg	− 12,332	00	8.88 × 10^–6^	2.94 × 10^–15^	1.16 × 10^–13^
	St. deviation	335.7988	0	0	3.52E−15	1.15E−13
Ant lion optimizer (ALO) [152]	Avg	− 1.61E+03	7.71E−06	3.73E−15	9.75E−12	2.00E−11
	St. deviation	3.14E+02	8.45E−06	1.50E−15	9.33E−12	1.13E−11
Spotted hyena optimizer (SHO) [46]	Avg	− 1.16E × 10^3^	0.00E+00	2.48E+000	3.68 × 10^–2^	9.29 × 10^–1^
	St. deviation	2.72E × 10^2^	0.00E+00	1.41E+000	1.15 × 10^–2^	9.52 × 10^–2^
Moth flame optimizer (MFO) [34]	Avg	− 8.500E+03	8.460E+01	1.260E+00	8.940E−01	1.160E−01
	St. deviation	7.260E+02	1.620E+01	7.300E−01	8.810E−01	1.930E−01
Harris hawks optimizer (HHO) [78]	Avg	− 12,561.38	0	8.88 × 10^–16^	8.92 × 10^–6^	0.000101
	St. deviation	40.82419	0	0	1.16 × 10^–5^	0.000132
Grey wolf optimizer (GWO) [149]	Avg	− 6.1200E+02	3.1100E−02	1.0600E−14	5.3400E−03	6.5400E−02
	St. deviation	− 4.0900E+02	4.740E+01	7.7800E−03	2.0700E−03	4.470E−03
Enhanced crow search algorithm (ECSA) [150]	Avg	− 2332.3867	0	8.88178E−16	0.11738407	0.444690657
	St. deviation	223.93995	0	0	0.2849633	0.199081675
Salp swarm algorithm (SSA) [148]	Avg	5.570E−02	0.000E+00	1.950E−01	1.420E−01	8.320E−02
	St. deviation	8.090E−01	0.000E+00	1.530E−01	5.570E−01	7.060E−01
Transient search optimization (TSO) [109]	Avg	− 12,569.5	00	8.88 × 10^–16^	1.30 × 10^–4^	7.55 × 10^–4^
	St. deviation	1.81 × 10^–2^	00	0	1.67 × 10^–4^	1.74 × 10^–3^
LF-SMA [8]	Avg	0.0004	− 3.2865	− 17.363	0.0130	1.07E−12
	St. deviation	5.89E−05	0.0536	2.1907	0.0090	2.29E−12
Proposed algorithm hSMA-SA	Avg	− 12,569.02623	**0**	8.88E−16	0.012678845	0.002689783
	St. deviation	0.43623993	0	0	0.012483005	0.001733379

### Evaluation of a few fixed-dimension functions

**Fig. 16:**
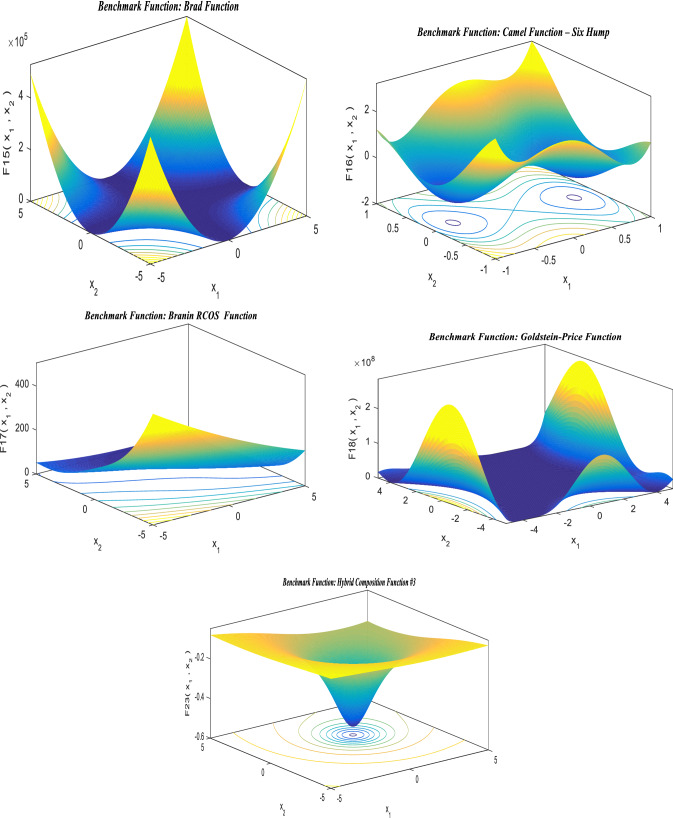
Fixed-dimension benchmark functions in 3D

**Fig. 17:**
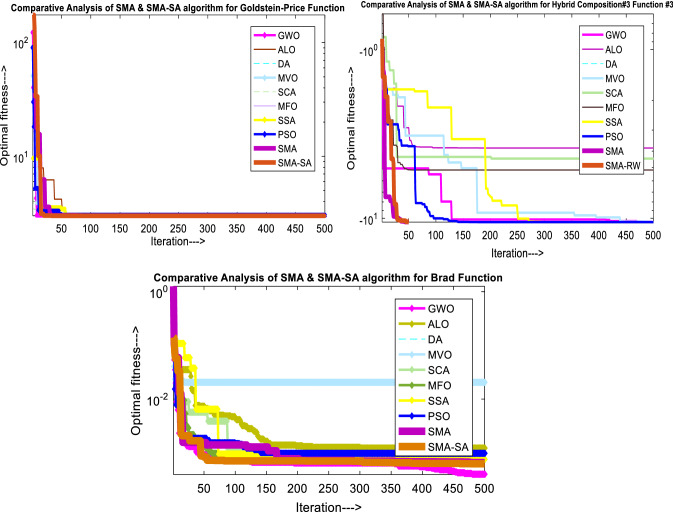
Convergence curve of hSMA-SA with known algorithms for fixed-dimension functions

**Fig. 18:**
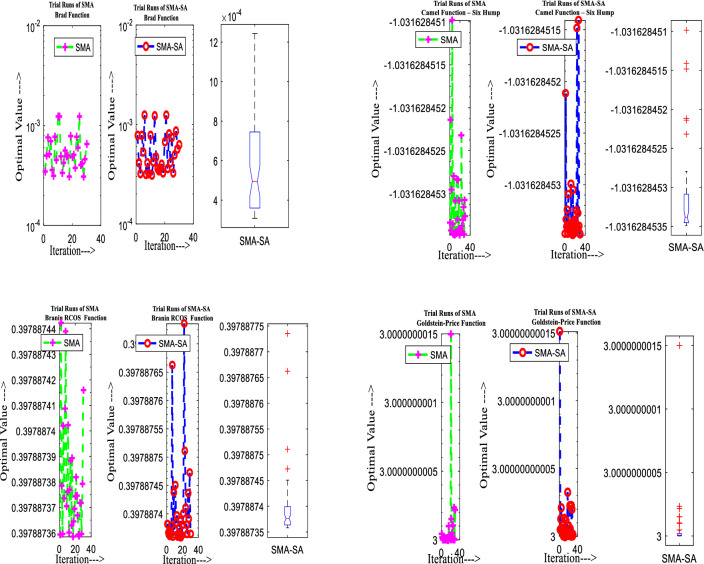
Trial runs of SMA and hSMA-SA for fixed-dimension functions

Figure 16 presents the characteristic curves of fixed-dimension functions and Fig. 17 showcases the comparison between hSMA-SA and other techniques. In this article, F15, F16, F17, F18, and F23 fixed-dimension benchmark functions are examined considering 30 trial runs with 500 iterations and the results are shown in Fig. 18. Using hSMA-SA, the simulation results for fixed-dimension functions are recorded in Table 12. The execution time for simulation process for fixed-dimension functions are recorded and tabled in Table 13. Table 14 contains the information of compared results of hSMA-SA algorithm with other already available metaheuristic search algorithms such as LSA [144], (SCA) [102], MFO [34], PSA [74], HHO-PS [50], (SSA) [148], SHO [46], GWO [149], HHO [78], MVO [24], ECSA [150], PSO [151], TSO [109], and ALO [152] considering standard deviation and average value. The results of fixed-dimension functions have a statistical analysis in selected points such as search record, convergence behavior, average fitness of population. The search record in the trail runs graph shows the positions of slime mould. The graph of convergence explains the variation in the location of slime mould during optimization procedure. The average fitness of the population describes the changes in the average population during whole optimization procedure. This better convergence certifies the effectiveness of the suggested algorithm. The statistical Wilcoxon rank sum test and *t* test were also used to confirm the suggested algorithm’s detailed behavior, and the low *p* value provided in Table 12 indicates that the produced algorithm has better convergence and is more effective. In addition, the null hypothesis is validated at a 95% level of significance by the *h* value. The parametric test of the suggested technique rejects the null hypothesis at the alpha significance level. If *h* = 1, the null hypothesis has been rejected at the alpha significance level. The alpha significance criterion did not permit a legitimate rejection of the null hypothesis if *h* = 0. It is noted from Fig. 17 that the results of F15, F16, F17, F18, and F23 fixed-dimension benchmark functions have improved convergence curves using hSMA-SA, justifying the aptness of the algorithm in verdict solutions for the fixed dimensions. The average and standard deviation outcomes reveal that hSMA-SA performs better for all five test functions than GWO, HHO-PS, SCA, PSO. It can be recorded from the convergence curves of Fig. 17 that hSMA-SA allows optimal convergences for a few test functions. From the comparison of convergence, it is well understood that hSMA-SA converges soon and catches the run as early as it attains the end condition. From the comparative curves of Fig. 17, it is noticeable that the proposed technique acts good in handling with F15, F16, and F23 and performs better than F17 and F18. Figure 18 reveals that the trial runs of the proposed hSMA-SA algorithm notably search in local and global space to find the optimal solution.

**Table 12:** Test outcomes of fixed-dimension functions using hSMA-SA technique

Function	Mean	Standard deviation	Best fitness value	Worst fitness value	Median	Wilcoxon rank sum test	*t* test	
						*p* value	*p* value	*h* value
Brad function (F15)	0.00057313	0.000284537	0.000308341	0.001243214	0.000443492	1.7344E−06	6.78516E−12	1
Camel function—six hump (F16)	− 1.031628453	5.42E−10	− 1.031628453	− 1.031628451	− 1.031628453	1.73E−06	7.16E−271	1
Branin RCOS function (F17)	0.397887411	8.70E−08	0.397887358	0.397887735	0.397887381	1.73E−06	6.34E−195	1
Goldstein-price function (F18)	3	2.77E−11	3	3	3	1.73E−06	0	1
Hybrid composition function #3 (F23)	− 8.732008933	3.082499131	− 10.52915056	− 5.172702813	− 10.49417343	0.25	0.039117859	1

**Table 13:** Execution time of fixed-dimension functions using hSMA-SA technique

Function	Best time	Average time	Worst time
Brad function (F15)	53.20313	56.91614583	65.640625
Camel function—six hump (F16)	43.98438	46.20729167	51.90625
Branin RCOS function (F17)	42.40625	44.60052083	47.453125
Goldstein-price function (F18)	20.79688	21.53385417	22.5625
Hybrid composition function #3 (F23)	0.078125	0.229166667	0.484375

**Table 14:** Benchmark functions with fixed dimensions compared with other techniques

Algorithm	Parameters	Fixed-dimension benchmark functions				
		Brad function (F15)	Camel function—six hump (F16)	Branin RCOS function (F17)	Goldstein-price function (F18)	Hybrid composition function #3 (F23)
Lightning search algorithm (LSA) [144]	Mean	0.024148546	0.000534843	− 1.031628453	3.000000000	− 7.910438367
	St. deviation	0.047279168	0.000424113	0.000000000	3.34499E−15	3.596042666
Enhanced crow search algorithm (ECSA) [150]	Mean	0.000327	− 1.03161	0.397993	3.00003	− 10.5359
	St. deviation	1.24337E−05	2.20378E−05	1.16E−04	2.752E−05	4.62E−04
Salp swarm algorithm (SSA) [148]	Mean	0.0000	0.1952	0.0000	0.1417	N/A
	St. deviation	0.0000	0.1527	0.0651	0.5571	N/A
Multi-verse optimizer (MVO) [24]	Mean	30.00705	50.00061	190.3	160.5312	N/A
	St. deviation	48.30615	52.70461	128.6659	158.2887	N/A
Transient search optimization (TSO) [109]	Mean	9.01 × 10^–4^	− 1.06 × 10^–1^	3.97 × 10^–1^	3.00E+000	10.5267
	St. deviation	1.06 × 10–4	2.86 × 10^–11^	2.46 × 10^–1^	9.05E+000	2.63 × 10^–2^
Particle swarm optimization (PSO) [151]	Mean	0.4081	0.6181	0.4694	0.3566	N/A
	St. deviation	0.8317	0.5347	0.8406	0.7841	N/A
Photon search algorithm (PSA) [74]	Mean	0.0077	− 1.036	0.3979	3	− 9.8189
	St. deviation	0.0224	2.33 × 10^–7^	1.41 × 10^–7^	1.36 × 10^–5^	1.8027
Sine–cosine algorithm (SCA) [102]	Mean	0.0230	0.0497	0.0000	0.0129	N/A
	St. deviation	0.0676	0.4921	0.1105	0.0134	N/A
Hybrid Harris hawks optimizer–pattern search algorithm (hHHO-PS) [50]	Mean	0.000307	− 1.03163	0.397887	3	− 10.5364
	St. deviation	1.65 × 10^–13^	1.11 × 10^–16^	00	2.63 × 10^–15^	7.69 × 10^–15^
Ant lion optimizer (ALO) [152]	Mean	14.56498	175.1532	316.0686	4.399206	N/A
	St. deviation	32.22876	46.50001	13.02047	1.66107	N/A
Spotted hyena optimizer (SHO) [46]	Mean	2.70 × 10^–3^	− 1.0316	0.398	3.000	− 1.68E+000
	St. deviation	5.43 × 10^–3^	5.78 × 10^–14^	1.26 × 10^–14^	2.66 × 10^–13^	2.64 × 10^–1^
Moth flame optimizer (MFO) [34]	Mean	66.73272	119.0146	345.4688	10.4086	N/A
	St. deviation	53.22555	28.3318	43.11578	3.747669	N/A
Harris hawks optimizer (HHO) [78]	Mean	0.00035	− 1.03163	0.397895	3.000001225	− 5.78398
	St. deviation	3.20 × 10^–5^	1.86 × 10^–9^	1.60 × 10^–5^	4.94 × 10^–6^	1.712458
Grey wolf optimizer (GWO) [149]	Mean	0.000337	− 1.03163	0.397889	3.000028	− 10.5343
	St. deviation	0.000625	− 1.03163	0.397887	3	− 8.55899
hSMA-SA-proposed algorithm	Mean	0.00057313	− 1.031628453	0.397887411	3	− 8.732008933
	St. deviation	0.000284537	5.42E−10	8.70E−08	2.77E−11	3.082499131

Thus, the outcomes for all benchmark functions are framed in Tables 6, 7, 8, 9, 10, 11, 12, 13, and 14 and the assessment of proposed hSMA-SA algorithm convergence curves for all benchmark functions are shown in Figs. 11, 14 and 17, and trial runs for all functions are depicted in Figs. 12, 15, and 18. The above result clearly shows that the proposed hSMA-SA algorithm is better than other algorithms. It also proves that hSMA-SA has proficient performance and very good convergence capability. As per the experiments carried out, the proposed hSMA-SA algorithm has given better results which can have balance between exploration and exploitation.

## Engineering-based optimization design problems

Eleven types of engineering-based optimization schemes are investigated to vote the usefulness of the recommended hSMA-SA algorithm. The hSMA-SA method is used to tackle these issues. As shown in Fig. 30, the findings for engineering design challenges were explored utilizing multiple metaheuristic search methods, with convergence curves compared to the classic SMA approach. Table 15 lists the engineering design problems, while Table 16 lists the fitness, average values, median values, standard deviation, as well as worst fitness values. Table 17 lists the Wilcoxon Rank sum test and *t* test values, while Table 18 lists the time of computation for engineering design issues.

**Table 15:** Special engineering designs

Special engineering function	I beam	Multiple disk clutch brake	Rolling element bearing	Spring design	Gear train	Speed reducer	Cantilever Beam	Three-bar truss	Pressure vessel	Welded beam	Belleville spring
Key objective	Minimize vertical deflection	Minimize weight	Maximize dynamic load	Minimize weight	Minimize gear ratio	Minimize weight	Minimize weight	Minimize weight	Minimize cost	Minimize cost	Minimize weight
Count of discrete variables	4	5	10	3	4	7	5	-	4	4	-
Count of constraint	4	8	9	4	1	11	1	3	4	7	5

**Table 16:** hSMA-SA outcomes of special engineering design problems

Name of design	Mean	Standard deviation	Best	Worst	Median
Spring design	0.013756625	0.001561454	0.012715329	0.017730689	0.012792095
Pressure vessel	6182.99867	451.5409611	5885.788679	7318.734233	5959.213246
Multiple disk clutch brake (discrete variables)	0.394509836	0.006702453	0.389654341	0.404666132	0.389665239
I beam design	0.00662596	3.62923E–09	0.006625958	0.006625976	0.006625959
Speed reducer problem	2994.491782	0.023016734	2994.474041	2994.595766	2994.486745
Cantilever beam design	1.303678864	0.000374902	1.303294886	1.305199129	1.303629652
Three-bar truss problem	270.2539599	2.361580897	264.2694671	273.4690636	270.9094455
Welded beam	1.778021239	0.143521546	1.725134404	2.321842966	1.728195083
Gear train	2.70E−11	7.34E−11	4.13E−16	3.10E−10	1.79E−12
Belleville spring	6.44E+22	7.81E+22	5.251751783	3.78E+23	5.69E+22
Rolling element bearing	− 85,525.79232	36.27683994	− 85,539.05618	− 85,346.80626	− 85,538.4479

**Table 17:** Parametric test outcomes using proposed hSMA-SA technique

Name of design	*p* value	*t* value	*h* value
Belleville spring	1.72E−06	9.71E−05	1
Pressure vessel	1.73E−06	9.20E−35	1
Spring design	1.73E−06	2.98E−29	1
I beam design	1.7344E−06	2.3546E−183	1
Multiple disk clutch brake (discrete variables)	1.73E−06	4.24E−53	1
Three-bar truss problem	1.73E−06	1.80E−61	1
Speed reducer problem	1.73E−06	4.36E−150	1
Rolling element bearing	1.73E−06	1.42E−99	1
Cantilever beam design	1.73E−06	1.81E−104	1
Gear train	1.73E−06	0.053538734	0
Welded beam	1.73E−06	1.65E−33	1

**Table 18:** Results recorded for time of computation using proposed hSMA-SA technique

Name of design	Best time	Mean time	Worst time
Pressure vessel	24.89063	25.81302083	26.90625
Speed reducer problem	29.5	31.37760417	34
Three-bar truss problem	24.6875	25.58489583	27.265625
Welded beam	28.35938	28.77760417	29.46875
Gear train	40.45313	43.490625	53.875
Belleville spring	54.70313	58.6578125	73.140625
Cantilever beam design	45.59375	47.33333333	49.0625
Rolling element bearing	31.92188	40.7046875	60.40625
I beam design	44.64063	47.19635417	52.578125
Spring design	24.95313	25.39947917	25.90625
Multiple disk clutch brake (discrete variables)	50.28125	52.92135417	56.953125

### Pressure vessel

**Fig. 19:**
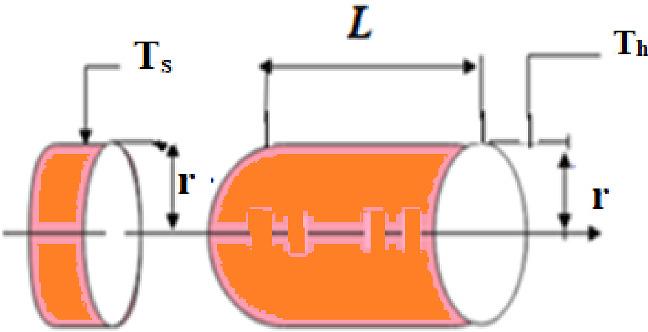
Design of pressure vessel

Figure 19 depicts the problem [153]. The fundamental goal of this challenge is to reduce construction costs. The problem has four factors and four parameters, which are (*t*_1_–*t*_4_): (*T*_s_) (*t*_1_, shell thickness), (*T*_h_) (*t*_2_, head’s thickness), *r* (*t*_3_, internal radius), and *L* (*t*_4_, unit’s length). This problem’s mathematical formula is represented in Eqs. (11) to (12d). Other optimization strategies were compared to the outcomes of using hSMA-SA to tackle this problem. The best results achieved by hSMA-SA with various optimization strategies are shown in Table 19. Furthermore, hSMA-SA outperforms known strategies handled this issue, and the outcomes provided by hSMA-SA are significantly superior to those obtained by other methodologies, based on these findings.

**Table 19:** Comparison of hSMA-SA results for pressure vessel optimization with known techniques

Competitive techniques	Optimal values for variables				Optimum cost
	*T*_s_	*T*_h_	*r*	*L*	
Suggested algorithm hSMA-SA	0.778348	0.3847859	40.328865	199.871477	5885.788679
BCMO [154]	0.7789243362	0.3850096372	40.3556904385	199.5028780967	6059.714
ChOA [52]	1.04375805524499	0.54814029437827	53.2363735879272	77.3302047573049	6.854064418325173+E
G-QPSO [155]	0.8125	0.4375	42.0984	176.6372	6059.7208
SMA [3]	0.7931	0.3932	40.6711	196.2178	5994.1857
ACO [156]	0.8125	0.4375	42.1036	176.5727	6059.0888
Branch-bound	1.125	0.625	47.7	117.701	8129.1
GWO [39]	0.8125	0.4345	42.0892	176.7587	6051.564
CDE [157]	0.8125	0.437500	42.098411	176.637690	6059.7340
AIS-GA [158]	0.8125	0.4375	42.098411	176.67972	6060.138
HHO-SCA [54]	0.945909	0.447138	46.8513	125.4684	6393.092794
HS [98]	1.099523	0.906579	44.456397	176.65887	6550.0230
DELC [159]	0.8125	0.4375	42.0984455	176.636595	6059.7143
SiC-PSO [160]	0.8125	0.4375	42.098446	176.636596	6059.714335
NPGA [161]	0.8125	0.437500	42.097398	176.654047	6059.946341
HHO [78]	0.8125	0.4375	42.098445	176.636596	6000.46259
CLPSO [162]	0.8125	0.4375	42.0984	176.6366	6059.7143
GeneAs [163]	0.9375	0.5000	48.3290	112.6790	6410.3811
GSA [20]	1.125	0.625	55.9887	84.4542	8538.84
Lagrangian multiplier	1.125	0.625	58.291	43.69	7198.043
MFO [34]	0.8125	0.4375	42.0981	176.641	6059.7143
MVO [24]	0.8125	0.4375	42.0907382	176.738690	6060.8066
SCA	0.817577	0.417932	41.74939	183.57270	6137.3724

We consider

11 $$ \vec{t} = \left[ {t_{1} t_{2} t_{3} t_{4} } \right] = \left[ {T_{{\text{s}}} T_{{\text{h}}} rL} \right] $$

To minimize,

12 $$ f(\vec{t}) = 0.6224t_{1} t_{3} t_{4} + 1.7781t_{2} t_{3}^{2} + 3.1661t_{1}^{2} t_{4} + 19.84t_{1}^{2} t_{3} $$

Here,

12a $$ G_{1} \left( {\vec{t}} \right) = - t_{1} + 0.0193t_{3} \le 0 $$

12b $$ G_{2} \left( {\vec{t}} \right) = t_{3} + 0.00954t_{3} \le 0 $$

12c $$ G_{3} \left( {\vec{t}} \right) = - \pi t_{3}^{2} t_{4} - \frac{4}{3}\pi t_{3}^{3} + 1296000 \le 0 $$

12d $$ G_{4} \left( {\vec{t}} \right) = t_{4} - 240 \le 0 $$

$$ \begin{gathered} {\text{Variable range}},0 \le t_{1} \le 99 \hfill \\ 0 \le t_{2} \le 99 \hfill \\ 10 \le t_{3} \le 200 \hfill \\ 10 \le t_{4} \le 20 \hfill \\ \end{gathered} $$

### Speed reducer

**Fig. 20:**
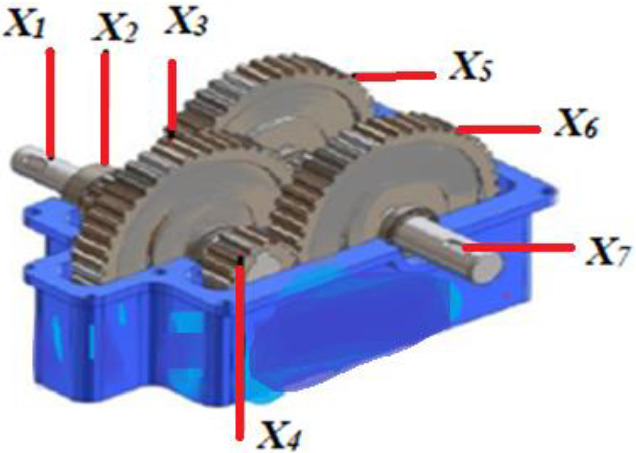
Design of speed reducer

This sort of issue comprises seven variables, as depicted in Fig. 20 [153]. It has a face width of (*b*_*w*_), a teeth module of (*t*_*m*_), a pinion teeth number of *x*, length among bearings for first shaft (*L*_*i*1_), length among bearings for second shaft (*L*_*i*2_), diameter of first shaft (*D*_*S*1_), and diameter of second shaft (*D*_*S*2_). First and foremost, the reducer weight to be lowered, which is the primary goal of this problem. The analysis’ findings are summarized in Table 19. The diagnostic answers of hSMA-SA are measured to those of GSA [20], HHO-SCA [54], PSO [164], OBSCA, MFO [34], SCA, HS [98], and GA [165]. The mathematics of the speed reducer optimization is framed in Eqs. (13) to (13k). The equations are written in the following format:

Reduce

13 $$ \begin{gathered} f(x) = 0.7854x_{1} x_{2} (3.3333x_{3}^{2} + 14.9334x_{3} - 43.0934)\hfill \\ - 1.508x_{1} (x_{6}^{2} + x_{7}^{2} )  + 7.4777(x_{6}^{3} + x_{7}^{3} ) + 0.7854(x_{4} x_{6}^{2} + x_{5} x_{7}^{2} ) \hfill \\ \end{gathered} $$

Subjected to

13a $$ g_{1} (\vec{x}) = \frac{27}{{b_{w} t_{m}^{2} x}} - 1 \le 0 $$

13b $$ g_{2} (\vec{x}) = \frac{397.5}{{b_{w} t_{m}^{2} x}} - 1 \le 0 $$

13c $$ g_{3} (\vec{x}) = \frac{{1.93D_{S1}^{3} }}{{t_{m} xD_{S1}^{4} }} - 1 \le 0 $$

13d $$ g_{4} (\vec{x}) = \frac{{1.93L_{i2}^{3} }}{{t_{m} xD_{S2}^{4} }} - 1 \le 0 $$

13e $$ g_{5} (\overrightarrow {x} ) = \frac{1}{{110D_{S1}^{3} }}\sqrt {\left( {\frac{{745.0L_{i1} }}{{t_{m} x}}} \right)^{2} } + 16.9 \times 10^{6} - 1 \le 0 $$

13f $$ g_{6} (\overrightarrow {x} ) = \frac{1}{{85D_{S2}^{3} }}\sqrt {\left( {\frac{{745.0L_{i2} }}{{t_{m} x}}} \right)^{2} } + 157.5 \times 10^{6} - 1 \le 0 $$

13g $$ g_{7} (\vec{x}) = \frac{{t_{m} x}}{40} - 1 \le 0 $$

13h $$ g_{8} (\vec{x}) = \frac{{5t_{m} }}{{b_{w} }} - 1 \le 0 $$

13i $$ g_{9} (\vec{x}) = \frac{{b_{w} }}{{12t_{m} }} - 1 \le 0 $$

13j $$ g_{10} (\vec{x}) = \frac{{1.5D_{S1} + 1.9}}{{12t_{m} }} - 1 \le 0 $$

13k $$ g_{11} (\vec{x}) = \frac{{1.1D_{S2} + 1.9}}{{L_{i2} }} - 1 \le 0 $$

Here,

$$ 2.6 \le b_{w} \le 3.6,0.7 \le t_{m} \le 0.8,17 \le x \le 28,7.3 \le L_{i1} \le 8.3,7.8 \le L_{i2} \le 8.3,2.9 \le D_{S1} \le 3.9\quad {\text{and}}\quad 5 \le D_{S2} \le 5.5 $$

### Three-bar truss engineering design

**Fig. 21:**
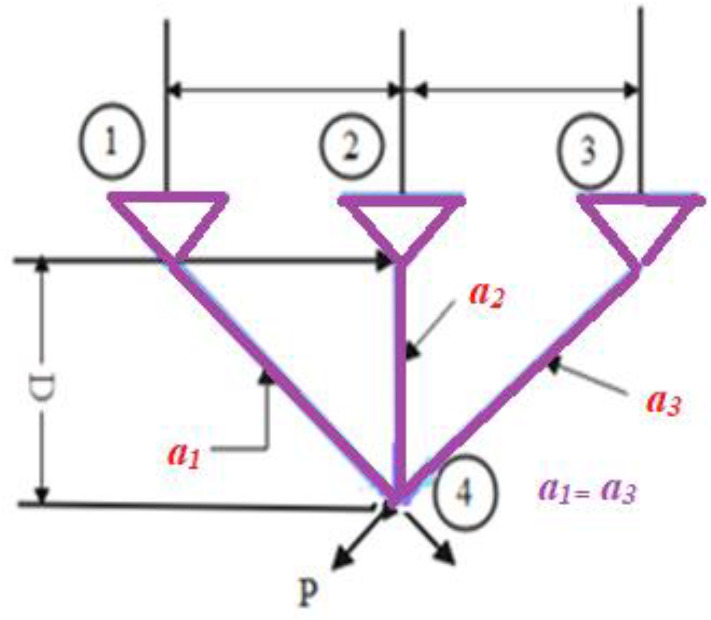
Three-bar truss engineering

The hSMA-SA technique is used to solve a 3-bar truss design issue; the relevant optimization issue is shown in Fig. 21. There are two variables and three parameters in this issue. The purpose of the truss plan is to lessen weight. Three constraints are present: deflection constraint, buckling constraint, as well as stress constraint. These three constraints are optimized to pull off the chosen goal. Equations (14–15c) quantitatively disclose the three-bar truss issue for various sorts of constraints. Table 20 compares the findings of hSMA-SA with those of other on-hand approaches. The suggested hSMA-SA algorithm appears to significantly improve the goal of weight loss:

14 $$ {\text{Consider}}\mathop x\limits^{ \to } = [x_{1} ,x_{2} ] = [a_{1} ,a_{2} ] $$

15 $$ {\text{Reduce}}\;f(\mathop x\limits^{ \to } ) = (2\sqrt 2 x_{1} + x_{2} ) \cdot l $$

15a $$ {\text{Subject to}}\;g_{1} (\mathop x\limits^{ \to } ) = \frac{{\sqrt 2 x_{1} + x_{2} }}{{\sqrt 2 x^{2}_{1} + 2x_{1} x_{2} }}P - \rho \le 0 $$

15b $$ g_{2} (\mathop x\limits^{ \to } ) = \frac{{x_{2} }}{{\sqrt 2 x^{2}_{1} + 2x_{1} x_{2} }}P - \rho \le 0 $$

15c $$ g_{3} (\mathop x\limits^{ \to } ) = \frac{1}{{\sqrt 2 x_{2} + x_{1} }}P - \rho \le 0 $$

$$ {\text{Variable}}\;{\text{range}}\quad 0 \le x_{1} ,x_{2} \le 1. $$

**Table 20:** Comparison of hSMA-SA results for speed reducer optimization with other techniques

Competitive techniques	Optimal values for variables							Optimum fitness
	*x*_1_	*x*_2_	*x*_3_	*x*_4_	*x*_5_	*x*_6_	*x*_7_	
Proposed hSMA-SA	3.5	0.7	17	7.3	7.715380	3.350218	5.286654	2994.474041
HS [98]	3.520124	0.7	17	8.37	7.8	3.366970	5.288719	3029.002
MFO [34]	3.507524	0.7	17	7.302397	7.802364	3.323541	5.287524	3009.571
GSA [20]	3.600000	0.7	17	8.3	7.8	3.369658	5.289224	3051.120
HHO-SCA [54]	3.506119	0.7	17	7.3	7.99141	3.452569	5.286749	3029.873076
GA [165]	3.510253	0.7	17	8.35	7.8	3.362201	5.287723	3067.561
PSO [164]	3.500019	0.7	17	8.3	7.8	3.352412	5.286715	3005.763
OBSCA	3.0879	0.7550	26.4738	7.3650	7.9577	3.4950	5.2312	3056.3122
SCA	3.508755	0.7	17	7.3	7.8	3.461020	5.289213	3030.563

where *l* = 100 cm, *P* = 2KN/cm^2^, *ρ* = 2 KN/cm^2^

### Welded beam

**Fig. 22:**
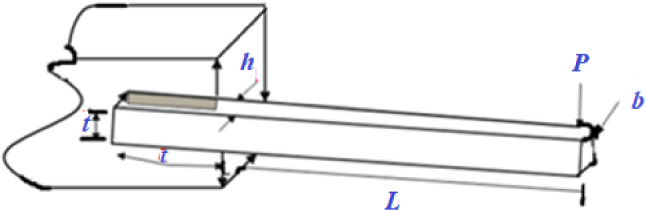
Welded mechanical beam model

This issue is represented in Fig. 22 [173]. The major goal is to reduce the production costs of the welded beam. (1) Bar height (*h*) is represented by *z*_4_, (2) weld thickness (*t*) is represented by *z*_3_, (3) bar length (*L*) is represented by *z*_2_, and (4) bar thickness (*b*) is represented by *z*_1_; these are the four variables that are subjected to buckling bar (Pc), end beam deflection (*d*), side restrictions and shear stress (*s*), and bending beam stress (*h*). Equations (16) to (18f) show the welded beam optimization design equations. The findings of hSMA-SA are measured with known techniques in Table 21. According to the investigation’s findings, the proposed approach is more capable of managing welded beam design with extreme precision (Table 22).

**Table 21:** Comparison of hSMA-SA results for three-bar truss optimization with known techniques

Competitive techniques	Optimal values for variables		Optimum weight
	*X*_1_	*X*_2_	
Proposed hSMA-SA	0.767861026	0.470855717	264.2694671
Ray and Liew [166]	0.788621037	0.408401334	263.8958466
Hernandez	0.788	0.408	263.9
CS [167]	0.789	0.409	263.972
Ray and Saini [168]	0.795	0.398	264.3
HHO-SCA [54]	0.788498	0.40875	263.8958665
Gandomi [169]	0.78867	0.40902	263.9716
CSA [170]	0.788638976	0.408350573	263.895844337
GWO-SA [171]	0.789	0.408	263.896
MBA [169]	0.789	0.409	263.896
WDE [52]	0.515535107819326	0.0156341500434795	2.639297829829848E+02
ALO [152]	0.789	0.408	263.8958434
DEDS [172]	0.789	0.408	263.896
Raj et al.	0.789764410	0.405176050	263.89671

**Table 22:** Comparison of hSMA-SA results for welded beam optimization design with known techniques

Competitive techniques	Optimal values for variables				Optimum cost
	*h*	*l*	*t*	*b*	
Proposed hSMA-SA	0.205727302	3.471126735	9.035476564	0.205781951	1.725134404
HS [98]	0.2442	6.2231	8.2915	0.2443	2.3807
PSO [164]	0.197411	3.315061	10.00000	0.201395	1.820395
Approx	0.2444	6.2189	8.2189	0.2444	2.3815
CDE [157]	0.203137	3.542998	9.033498	0.206179	1.733462
David	0.2434	6.2552	8.2915	0.2444	2.3841
GSA [20]	0.1821	3.857	10	0.2024	1.88
(PSOStr) [174]	0.2015	3.526	9.041398	0.205706	1.731186
HHO-SCA [54]	0.190086	3.696496	9.386343	0.204157	1.779032249
MFO [34]	0.203567	3.443025	9.230278	0.212359	1.732541
Gandomi et al. (FA) [175]	0.2015	3.562	9.0414	0.2057	1.73121
SCA	0.204695	3.536291	9.004290	0.210025	1.759173

Let us consider

16 $$ \vec{z} = \left[ {z_{1} z_{2} z_{3} z_{4} } \right] = \left[ {hltb} \right]. $$

17 $$ f(\vec{z}) = 1.10471z_{1}^{2} z_{2} + 0.04811z_{3} z_{4} \left( {14.0 + z_{2} } \right) $$

By addressing,

17a $$ g_{1} (\vec{z}) = \tau (\vec{z}) - \tau_{{{\text{M}}axi}} \le 0, $$

17b $$ g_{2} (\vec{z}) = \rho (\vec{z}) - \rho_{{{\text{Max}}i}} \le 0 $$

17c $$ g_{3} (\vec{z}) = \delta (\vec{z}) - \delta_{{{\text{Max}}i}} \le 0 $$

17d $$ g_{4} (\vec{z}) = z_{1} - z_{4} \le 0 $$

17e $$ g_{5} (\vec{z}) = P_{i} - P_{c} (\vec{z}) \le 0 $$

17f $$ g_{6} (\vec{z}) = 0.125 - z_{1} \le 0 $$

17g $$ g_{7} (\vec{z}) = 1.10471z_{1}^{2} + 0.04811z_{3} z_{4} (14.0 + z_{2} ) - 5.0 \le 0 $$

Range of variables: $0.1 \le z_{1} \le 2,0.1 \le z_{2} \le 10,0.1 \le z_{3} \le 10,0.1 \le z_{4} \le 2.$

Here,

18a $$ \tau (\vec{z}) = \sqrt {(\tau^{/} )^{2} + 2\tau^{/} \tau^{//} \frac{{z_{2} }}{2R} + (\tau^{//} )^{2} ,} $$

18b $$ \tau^{/} = \frac{{P_{i} }}{{\sqrt 2 z_{1} z_{2} }},\tau^{//} = \frac{MR}{J},M = P_{i} \left( {L + \frac{{z_{2} }}{2}} \right), $$

18c $$ R = \sqrt {\frac{{z_{2}^{2} }}{4} + \left( {\frac{{z_{1} + z_{3} }}{2}} \right)^{2} } $$

18d $$ J = 2\left\{ {\sqrt 2 z_{1} z_{2} \left[ {\frac{{z_{2}^{2} }}{4} + \left( {\frac{{z_{1} + z_{3} }}{2}} \right)^{2} } \right]} \right\} $$

18e $$ \rho (\vec{y}) = \frac{{6P_{i} L}}{{z_{4} z_{3}^{2} }},\delta (\vec{y}) = \frac{{6P_{i} L^{3} }}{{Ez_{2}^{2} z_{4} }} $$

18f $$ P_{c} (\vec{z}) = \frac{{4.013E\frac{{\sqrt {z_{3}^{2} z_{4}^{6} } }}{36}}}{{L^{2} }}\left( {1 - \frac{{z_{3} }}{2L}\sqrt{\frac{E}{4G}}  } \right) $$

$$ \begin{gathered} L = 14in,\delta_{Maxi} = 0.25in,E = 30 \times 1^{6} psi,G = 12 \times 10^{6} psi, \hfill \\ \tau_{Maxi} = 13600psi,\rho_{Maxi} = 3000psi,P = 6000lb \hfill \\ \end{gathered} $$

### Gear train design

**Fig. 23:**
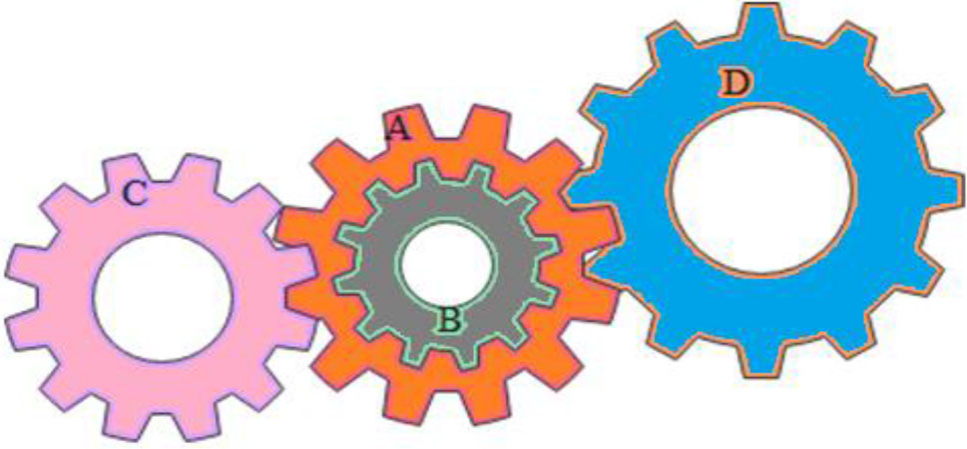
Gear train optimization design

This is one of the various engineering difficulties that comprise four variables and a tooth ratio, as spotted in Fig. 23 [153]. The architectural design’s general purpose is to help reduce the scalar value of the gears and the teeth ratio as much as feasible. As an outcome, each gear’s teeth are handled as design variables throughout the process. Table 23 shows the analytical results for a comparison of hSMA-SA with other methodologies. The suggested method is more successful in determining the gear train ratio, according to the observations. The following is a model for the required formulas:

**Table 23:** Comparison of hSMA-SA results for gear train optimization design with known techniques

Competitive techniques	Optimal values for variables				Gear ratio	Optimum fitness
	*x*_1_ (*T*_*d*_)	*x*_2_ (*T*_*b*_)	*x*_3_ (*T*_*a*_)	*x*_4_ (*T*_*f*_)		
Proposed hSMA-SA	17.39759773	12.00546725	12	57.39404929	NA	2.70E−11
IMFO [176]	19	14	34	50	NA	3.0498E−13
MARS [177]	19	16	43	49	0.1442	2.7E−12
CSA [167]	19.000	16.000	43.000	49.000	NA	2.7008571489E−12
ISA [178]	19	16	43	49	NA	2.701E−12
HGA [179]	15	21	59	37	NA	3.07E−10
MIBBSQP [180]	18	22	45	60	0.146666	5.7E−06
MP [181]	18	22	45	60	0.1467	5.712E−06
Ahga1 [179]	13	24	47	46	NA	9.92E−10
IDCNLP [182]	14	29	47	59	0.146411	4.5E−06
MBA [169]	16	19	49	43	0.1442	2.7005E−0.12
MINSLIP [180]	19	16	42	50	NA	2.33E−07
Ahga2 [179]	13	20	53	34	NA	2.31E−11
ALO [152]	19.00	16.00	43.00	49.00	NA	2.7009E−012
CAPSO [169]	16	19	49	43	0.1442	2.701E−12

Let us consider

19 $$ {\vec{\text{G}}\text{et}} = \left[ {{\text{Get}}_{1} {\text{Get}}_{2} {\text{Get}}_{3} {\text{Get}}_{4} } \right] = \left[ {M_{A} M_{B} M_{C} M_{D} } \right]. $$

To minimize,

19a $$ f(\overrightarrow {{{\text{Get}}}} ) = \left( {\frac{1}{6.931} - \frac{{{\text{Get}}_{3} {\text{Get}}_{4} }}{{{\text{Get}}_{1} {\text{Get}}_{4} }}} \right)^{2} $$

Subjected to

19b $$ 12 \le {\text{Get}}_{1} ,{\text{Get}}_{2} ,{\text{Get}}_{3} ,{\text{Get}}_{4} \le 60 $$

### Belleville spring

**Fig. 24:**
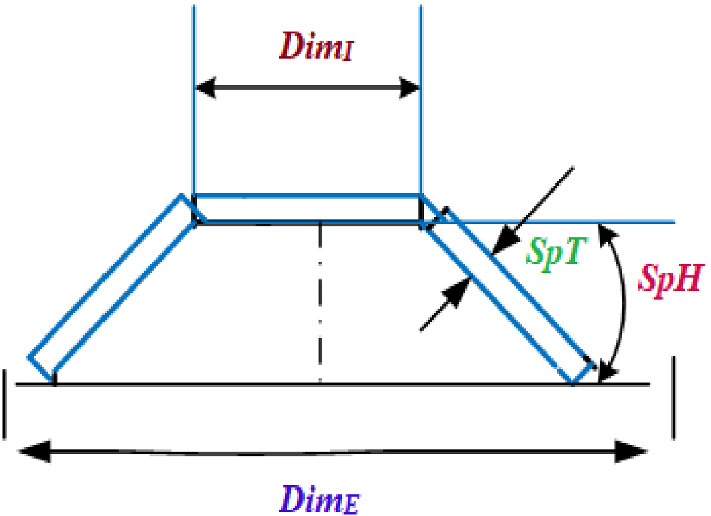
Belleville spring engineering design

Figure 24 depicts this problem. This is a method for reducing the issue by picking a parameter that already survives in the defined variable ratio limits. The major goal of this task is to reduce the weight while staying within the limitations. Deflection, deflection height, the interior and exterior portions of the diameter, compressive forms of stresses, and slope will all be altered when the limitations are applied. The spring height (SpH), exterior part diameter (Dim_E_), internal part diameter (Dim_I_), and Belleville spring thickness (SpT) of a Belleville spring are all built with minimal weight. The comparative findings are shown in Table 24. The recommended technique is more successful in solving the spring design challenge, according to observations. The formulae are listed as

20 $$ {\text{Minimizing}}; f(w) = 0.07075\pi ({\text{Dim}}_{E}^{2} - {\text{Dim}}_{I}^{2} )t. $$

**Table 24:** Comparison of hSMA-SA results for Belleville spring optimization with known techniques

Competitive techniques	Optimal values for variables				Optimum fitness
	*W*_1_	*W*_2_	*W*_3_	*W*_4_	
Suggested hSMA-SA	12.01	8.242835383	0.309690187	0.2	5.251751783
HHO-SCA [54]	11.98603	10.0002	0.204206	0.2	1.98170396
TLBO [40]	12.01	10.03047	0.204143	0.2	0.198966
MBA [169]	12.01	10.030473	0.204143	0.2	0.198965

Subjected to

21 $$ b_{1} (w) = G - \frac{{4P\lambda_{{{\text{MaX}}}} }}{{(1 - \delta^{2} )\alpha {\text{Dim}}_{E} }}\left[ {\delta \left( {{\text{SpH}} - \frac{{\lambda_{{{\text{MaX}}}} }}{2}} \right) + \mu t} \right] \ge 0. $$

21a $$ b_{2} (w) = \left( {\frac{{4P\lambda_{{{\text{MaX}}}} }}{{(1 - \delta^{2} )\alpha {\text{Dim}}_{E} }}\left[ {\left( {{\text{SpH}} - \frac{{\lambda_{{}} }}{2}} \right)({\text{SpH}} - \lambda )t + t^{3} } \right]} \right)\lambda_{{{\text{MaX}}}} - P_{{{\text{MaX}}}} \ge 0 $$

21b $$ b_{3} (w) = \lambda_{1} - \lambda_{MaX} \ge 0 $$

21c $$ b_{4} (w) = H - {\text{SpH}} - t \ge 0 $$

21d $$ b_{5} (w) = {\text{Dim}}_{{{\text{MaX}}}} - {\text{Dim}}_{E} \ge 0 $$

21e $$ b_{6} (w) = {\text{Dim}}_{E} - {\text{Dim}}_{I} \ge 0 $$

21f $$ b_{7} (w) = 0.3 - \frac{{{\text{SpH}}}}{{{\text{Dim}}_{E} - {\text{Dim}}_{I} }} \ge 0, $$

where

$$ \alpha = \frac{6}{\pi \ln J}\left( {\frac{J - 1}{{\ln J}} - 1} \right)^{2} $$

$$ \delta = \frac{6}{\pi \ln J}\left( {\frac{J - 1}{{\ln J}} - 1} \right) $$

$$ \mu = \frac{6}{\pi \ln J}\left( {\frac{J - 1}{2}} \right) $$

*P*_MaX_ = 5400 lb.

*P* = 30e6 psi, $\lambda_{{{\text{MaX}}}}$ = 0.2 in, $\delta$ = 0.3, *G* = 200 Kpsi, *H* = 2 in, Dim_MAX_ = 12.01 in, $J = \frac{{{\text{Dim}}_{E} }}{{{\text{Dim}}_{I} }}$, $\lambda_{1} = f(a)a,\;a = \frac{{{\text{SpH}}}}{t}.$

### Cantilever beam design

**Fig. 25:**
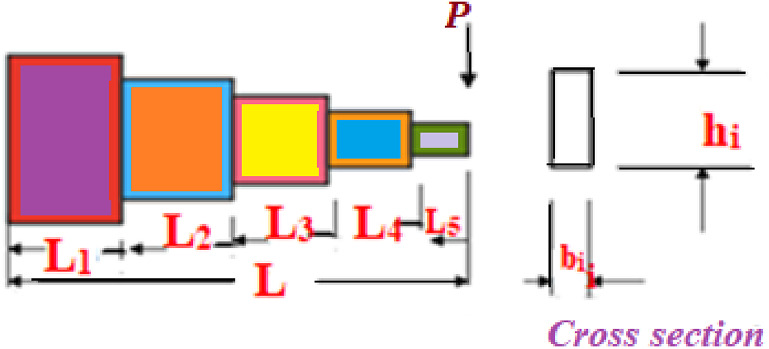
Design of cantilever beam

The goal of this civil engineering task, as shown in Fig. 25, is to reduce beam weight as much as possible. There are five distinct types of forms in this problem [173]. As seen in Eq. (22), the key objective is to pull down the beam’s weight. The complete design configuration consists of structural features of five elements that must be kept unchanged, with the beam thickness remaining constant, in order to reach the ultimate optimal solution, which is illustrated by Eqs. (33–34). The findings are compared to those obtained using various approaches in Table 25. hSMA-SA outperformed other approaches in terms of beam weight reduction. The mathematics are shown as follows:

$$ {\text{Let us consider}},\;{\vec{\text{L}}\text{en}} = [{\text{Len}}_{1} {\text{Len}}_{2} {\text{Len}}_{3} {\text{Len}}_{4} ] $$

22 $$ f({\vec{\text{L}}\text{en}}) = 0.6224({\text{Len}}_{1} + {\text{Len}}_{2} + {\text{Len}}_{3} + {\text{Len}}_{4} + {\text{Len}}_{5} ). $$

**Table 25:** Comparison of hSMA-SA outcomes for cantilever beam optimization with known techniques

Competitive techniques	Optimal values for variables					Optimum weight
	*L*_1_	*L*_2_	*L*_3_	*L*_4_	*L*_5_	
Proposed hSMA-SA	5.982032535	4.846178775	4.491073327	3.48171237	2.138830846	1.303294886
HHO-PS [50]	5.978829	4.876628	4.464572	3.479744	2.139358	1.303251
IMFO [176]	5.97822	4.87623	4.46610	3.47945	2.13912	1.30660
SMA [3]	6.017757	5.310892	4.493758	3.501106	2.150159	1.339957
GCA_I [24]	6.0100	5.3000	4.4900	3.4900	2.1500	1.3400
GWO-SA [171]	5.9854	4.87	4.4493	3.5172	2.1187	1.3033
MMA [183]	6.0100	5.3000	4.4900	3.4900	2.1500	1.3400
MVO [24]	6.02394022154	5.30301123355	4.4950113234	3.4960223242	2.15272617	1.3399595
CS [184]	6.0089	5.3049	4.5023	3.5077	2.1504	1.33999
SOS [185]	6.01878	5.30344	4.49587	3.49896	2.15564	1.33996
HHO-SCA [54]	5.937725	4.85041	4.622404	3.45347	2.089114	1.30412236

By addressing,

23 $$ g({\vec{\text{L}}\text{en}}) = \frac{61}{{{\text{Len}}_{1}^{3} }} + \frac{37}{{{\text{Len}}_{2}^{3} }} + \frac{19}{{{\text{Len}}_{3}^{3} }} + \frac{7}{{{\text{Len}}_{4}^{3} }} + \frac{1}{{{\text{Len}}_{5}^{3} }} \le 1 $$

Ranges of variables are $0.01 \le {\text{Len}}_{1} ,{\text{Len}}_{2} ,{\text{Len}}_{3} ,{\text{Len}}_{4} ,{\text{Len}}_{5} \le 100$.

### Rolling element bearing

**Fig. 26:**
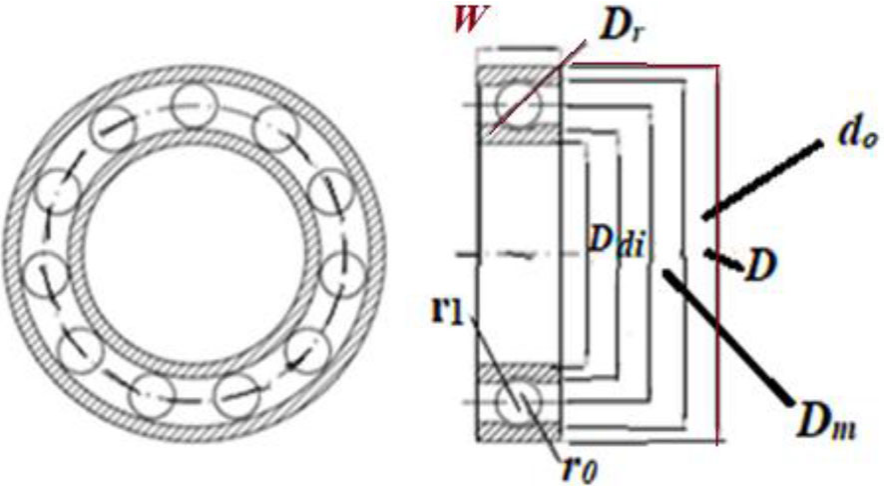
Design of rolling bearing

The target is to augment the bearing capability of the rolling element, as indicated in Fig. 26 [186]. The engineering design issue comprises a total of 10 decision variables that are used to determine the best bearing design for increasing load-carrying capacity. (1) Diameter of pitch (Dim_P_), (2) diameter of Ball (Dim_B_), (3) ball numbers (*N*_B_), (4) curvature coefficient of the outer raceway, and (5) curvature coefficient of inner raceway are the five variables that are given substantial importance, while the remaining five variables (KD_Min_, KD_Max_, *ε*, *e*, and *f*) are only assessed for discrete integers and influence interior section of the geometry circuitously. The findings of hSMA-SA are compared with various approaches for this problem in Table 26. Equations (24a through 25c) show the mathematical equations for this design challenge.

**Table 26:** Comparison of hSMA-SA results for rolling element beam optimization with known techniques

Competitive algorithms	Optimal values for variables										Optimum fitness
	*r*_1_	*r*_2_	*r*_3_	*r*_4_	*r*_5_	*r*_6_	*r*_7_	*r*_8_	*r*_9_	*r*_10_	
Proposed hSMA-SA	125.7224792	21.42329479	11.00114251	0.515	0.515000037	0.471814754	0.615593477	0.300001854	0.098010023	0.607557895	− 85,539.05618
SHO [46]	125	21.40732	10.93268	0.515	0.515	0.4	0.7	0.3	0.2	0.6	85,054.532
HHO [78]	125.00	21.00	11.092073	0.51500	0.51500	0.4000	0.6000	0.3000	0.050474	0.600	83,011.88329
WCA [187]	125.721167	21.42300	1.001030	0.515000	0.515000	0.401514	0.659047	0.300032	0.040045	0.600000	85,538.48
PVS [188]	125.719060	21.425590	11.000000	0.515000	0.515000	0.400430	0.680160	0.300000	0.079990	0.700000	81,859.741210
SCA [167]	125	21.03287	10.96571	0.515	0.515	0.5	0.7	0.3	0.027780	0.62912	83,431.117
MFO [34]	125	21.03287	10.96571	0.515	0.515000	0.5	0.67584	0.300214	0.02397	0.61001	84,002.524
MVO [24]	125.6002	21.32250	10.97338	0.515	0.515000	0.5	0.68782	0.301948	0.03617	0.61061	84,491.266

For maximizing,

24a $$ C_{{\text{D}}} = f_{{\text{C}}} N^{2/3} {\text{Dim}}_{{\text{B}}}^{1.8} . $$

If $${\text{DIM}} \le 25.4\;{\text{mm}}$$

24b $$ C_{{\text{D}}} = 3.647f_{{\text{C}}} N^{2/3} {\text{Dim}}_{{\text{B}}}^{1.4} $$

If $${\text{Dim}} \ge 25.4\;{\text{mm}}$$.

Addressing,

25 $$ r_{1} (y) = \frac{{\theta_{0} }}{{2\sin^{ - 1} \left( {\frac{{{\text{Dim}}_{{\text{B}}} }}{{{\text{Dim}}_{{{\text{MaX}}}} }}} \right)}} - N + 1 \ge 0 $$

25a $$ r_{2} (y) = 2{\text{Dim}}_{{\text{B}}} - K_{{{\text{Dim}}_{{{\text{MIN}}}} }} ({\text{DIM}} - \dim ) \ge 0 $$

25b $$ r_{3} (y) = K_{{{\text{DIM}}_{{{\text{MAX}}}} }} ({\text{DIM}} - \dim ) \ge 0 $$

25c $$ r_{4} (y) = \beta B_{{\text{W}}} - {\text{Dim}}_{{\text{B}}} \le 0 $$

25d $$ r_{5} (y) = {\text{DIM}}_{{{\text{MaX}}}} - 0.5({\text{DIM}} + \dim ) \ge 0 $$

25e $$ r_{6} (y) = {\text{DIM}}_{{{\text{MaX}}}} - 0.5({\text{DIM}} + \dim ) \ge 0 $$

25f $$ r_{7} (y) = (0.5 + {\text{re}})({\text{DIM}} + \dim ) \ge 0 $$

25g $$ r_{8} (y) = 0.5({\text{DIM}} - {\text{DIM}}_{{{\text{MaX}}}} - {\text{DIM}}_{{\text{B}}} ) - \alpha {\text{DIM}}_{{\text{B}}} \ge 0 $$

25h $$ r_{9} (y) = f_{I} \ge 0.515 $$

25i $$ r_{10} (y) = f_{0} \ge 0.515 $$

Here,

$$\begin{aligned} f_{{\text{c}}} &= 37.91\left[ {1 + \left\{ {1.04\left( {\frac{1 - \varepsilon }{{1 + \varepsilon }}} \right)^{1.72} \left( {\frac{{f_{I} \left( {2f_{0} - 1} \right)}}{{f_{0} \left( {2f_{I} - 1} \right)}}} \right)^{0.41} } \right\}^{10/3} } \right]^{ - 0.3}\\ &\quad \times \left[ {\frac{{\varepsilon^{0.3} \left( {1 - \varepsilon } \right)^{1.39} }}{{\left( {1 + \varepsilon } \right)^{1/3} }}} \right]\left[ {\frac{{2f_{I} }}{{2f_{I} - 1}}} \right]^{0.41} \end{aligned}$$

$$ \begin{aligned}
  {\theta _0} &  = 2\pi  - 2{\cos ^{ - 1}} \\ 
   & \quad  \times \left( {\frac{{\begin{array}{*{20}{c}}
  {[{{\left\{ {({\text{DIM}} - \dim )/2 - 3(t/4)} \right\}}^2} + {{\left( {{\text{DIM}}/2 - t/4 - {\text{DI}}{{\text{M}}_{\text{B}}}} \right)}^2}} \\ 
  { - {{\left\{ {\dim /2 + t/4} \right\}}^2}]} 
\end{array}}}{{2\left\{ {({\text{DIM}} - \dim )/2 - 3(t/4)} \right\}\left\{ {D/2 - t/4 - {\text{DI}}{{\text{M}}_{\text{B}}}} \right\}}}} \right)  
\end{aligned} $$

$$ \varepsilon = \frac{{{\text{DIM}}_{B} }}{{{\text{DIM}}_{{{\text{MAX}}}} }},\quad f_{I} = \frac{{R_{I} }}{{{\text{DIM}}_{{\text{B}}} }},\quad f_{0} = \frac{{R_{0} }}{{{\text{DIM}}_{{\text{B}}} }},\quad t = {\text{DIM}} - \dim - 2{\text{DIM}}_{{\text{B}}} $$

$$ {\text{DIM}} = 160,\quad \dim = 90,\quad B_{{\text{W}}} = 30,\quad R_{I} = R_{0} = 11.033 $$

$$ 0.5\left( {{\text{DIM}} + \dim } \right) \le {\text{DIM}}_{{{\text{MAX}}}} \le 0.6\left( {{\text{DIM}} + \dim } \right),0.15\left( {{\text{DIM}} - \dim } \right) \le {\text{DIM}}_{{\text{B}}} \le 0.45\left( {{\text{DIM}} - \dim } \right),4 \le N \le 50 $$

$$0.515 \le f_{I}$$ and $$f_{0} \le 0.6$$

$$\begin{aligned} 0.4 &\le K_{{{\text{DIM}}_{{{\text{MIN}}}} }} \le 0.5,0.6 \le K_{{{\text{DIM}}_{{{\text{MAX}}}} }} \le 0.7,\\ 0.3 &\le {\text{re}} \le 0.1,0.02 \le {\text{re}} \le 0.1,0.6 \le \beta \le 0.85.\end{aligned} $$

#### I beam design

**Fig. 27:**
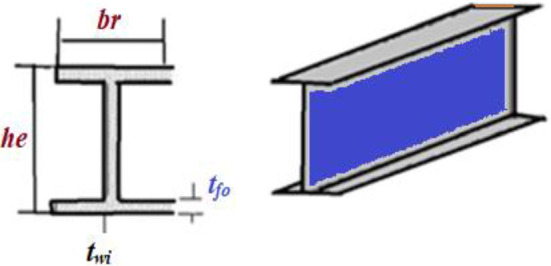
I beam structure

Essentially, the problem tries to reduce the vertical I beam deviation by changing the four parameters of the vertical I beam. Figure 27 depicts the four parameters br, he, *t*_wi_, and *t*_fo_. According to [189], in array to achieve the proportions of the beam indicated in the picture, geometric and strength constraints must be satisfied in order to optimize using the following criteria: (1) when a beam is displaced by applying force, its cross-section lowers its volume for a given length. (2) When the beam is moved by applying force, there is a static deflection to be recorded. In Eqs. (26–28), the mathematical formulas are given. The investigative outcomes of hSMA-SA are measured with other well-known procedures in Table 27.

**Table 27:** Comparison of hSMA-SA results for I beam optimization with known techniques

Competitive techniques	Optimal values for variables				Optimum fitness
	(br) × 1	(he) × 2	(*t*_wi_) × 3	(*t*_fo_) × 4	
Proposed hSMA-SA	50	80	1.764705807	5	0.006625958
BWOA [190]	50.00	80.00	1.76470588	5.00	0.00625958
SMA [3]	49.998845	79.994327	1.764747	4.999742	0.006627
HHO-PS [50]	50.00	80.00	1.764706	5.00	0.006626
CS [184]	50.0000	80.0000	0.9000	2.3217	0.0131
MFO [34]	50.000	80.000	1.7647	5.000	0.0066259
SOS [185]	50.0000	80.0000	0.9000	2.3218	0.0131
CSA [167]	49.99999	80	0.9	2.3217923	0.013074119
ARMS [191]	37.05	80	1.71	2.31	0.131
Improved ARMS [191]	48.42	79.99	0.9	2.4	0.131

Consider

26 $$ \vec{x} = [\begin{array}{*{20}c} {x_{1} } & {x_{2} } & {x_{3} } & {x_{4} } & {x_{5} ]} \\ \end{array} = [\begin{array}{*{20}c} {{\text{br}}} & {{\text{he}}} & {t_{{{\text{wi}}}} } & {t_{{{\text{fo}}}} } \\ \end{array} ], $$

27 $$ {\text{Minimize}}\;f(\vec{x}) = \frac{5000}{{\frac{{t{}_{{{\text{wi}}}}({\text{he}} - 2t_{{{\text{fo}}}} )^{3} }}{12} + \frac{{{\text{br}}t_{{{\text{fo}}}}^{3} }}{6} + 2{\text{br}}t_{{{\text{fo}}}} \left( {\frac{{{\text{he}} - t_{{{\text{fo}}}} }}{2}} \right)^{2} }}, $$

28 $$ {\text{Subjected to}}\;g(x) = 2{\text{br}}t_{{{\text{wi}}}} + t_{{{\text{wi}}}} ({\text{he}} - 2t_{{{\text{fo}}}} ) \le 0, $$

Variable range $$10 \le x_{1} \le 50,10 \le x_{2} \le 80,0.9 \le x_{3} \le 5,0.9 \le x_{4} \le 5.$$

#### Tension/compression spring design problem

**Fig. 28:**
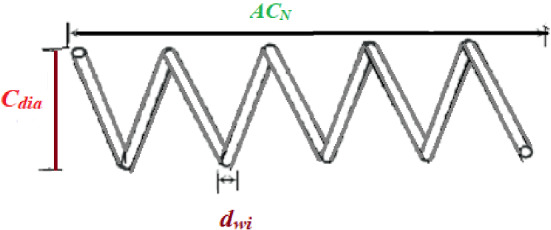
Spring engineering tension/compression design

This issue is indicated in Fig. 28 and is part of the mechanical engineering problem [173]. The key feature of the plan is that it minimizes the spring weight. Three sorts of variable designs are required to address this problem: diameter of wire (*d*_wi_), diameter of mean coil (*C*_dia_), and active coil number (AC_*N*_). The size of the surge, the minimum variation, and the shear stress constraints will all have a part in the design. The numerical for this problem are shown in Eqs. (29)–(30d). When the findings of hSMA-SA are compared to those of other approaches, as shown in Table 28, it is clear that hSMA-SA effectively decreases the spring’s weight by a little amount.

**Table 28:** Comparison of hSMA-SA results for the spring engineering tension/compression with known techniques

Competitive techniques	Optimal values for variables			Optimum weight
	*d*_wi_	*C*_dia_	AC_*N*_	
Proposed hSMA-SA	0.050058749	0.3187474	13.91919024	0.012715329
GA [165]	0.05010	0.310111	14.0000	0.013036251
PSO [164]	0.05000	0.3140414	15.0000	0.013192580
DELC [159]	0.051689061	0.356717741	11.28896566	0.012665233
IMFO [176]	0.051688973	0.356715627	11.289089342	0.012665233
AIS-GA	0.0516608	0.3560323	11.329555	0.0126666
HS [98]	0.05025	0.316351	15.23960	0.012776352
HHO-SCA [54]	0.054693	0.433378	7.891402	0.012822904
CDE [157]	0.051609	0.354714	11.410831	0.0126702
G-QPSO [155]	0.051515	0.352529	11.538862	0.012665
GSA [20]	0.05000	0.317312	14.22867	0.012873881
BCMO [154]	0.0516597413	0.3560124935	11.3304429494	0.012665
SCA [167]	0.050780	0.334779	12.72269	0.012709667
MALO [192]	0.051759	0.358411	11.191500	0.0126660
MVO [24]	0.05000	0.315956	14.22623	0.012816930
HHO-PS [50]	0.051682	0.356552	11.29867	0.012665
MFO [34]	0.05000	0.313501	14.03279	0.012753902
VCS [193]	0.051685684299756	0.356636508703361	11.29372966824506	0.012665222962643
BRGA	0.05167471	0.35637260	11.3092294	0.012665237
WCA [187]	0.051680	0.356522	11.300410	0.012665
MBA [169]	0.051656	0.355940	11.344665	0.012665
HEAA	0.0516895376	0.3567292035	11.288293703	0.012665233

Let us consider

29 $$ {\vec{\text{S}}\text{p}} = \left[ {{\text{Sp}}_{1} {\text{Sp}}_{2} {\text{Sp}}_{3} } \right] = \left[ {d_{{{\text{wi}}}} C_{{{\text{dia}}}} {\text{AC}}_{N} } \right]. $$

But to minimize,

30 $$ f({\vec{\text{S}}\text{p}}) = \left( {{\text{Sp}}_{3} + 2} \right){\text{Sp}}_{2} {\text{Sp}}_{1}^{2} $$

30a $$ g_{1} ({\vec{\text{S}}\text{p}}) = 1 - \frac{{{\text{Sp}}_{2}^{3} {\text{Sp}}_{3} }}{{71785{\text{Sp}}_{1}^{4} }} \le 0 $$

30b $$ g_{2} ({\vec{\text{S}}\text{p}}) = \frac{{4{\text{Sp}}_{2}^{2} - {\text{Sp}}_{1} {\text{Sp}}_{2} }}{{12566\left( {{\text{Sp}}_{2} {\text{Sp}}_{1}^{3} - {\text{Sp}}_{1}^{4} } \right)}} + \frac{1}{{5108{\text{Sp}}_{1}^{2} }} \le 0 $$

30c $$ g_{3} ({\vec{\text{S}}\text{p}}) = 1 - \frac{{140.4{\text{Sp}}_{1} }}{{{\text{Sp}}_{2}^{2} {\text{Sp}}_{3} }} \le 0 $$

30d $$ g_{4} ({\vec{\text{S}}\text{p}}) = \frac{{{\text{Sp}}_{1} + {\text{Sp}}_{2} }}{1.5} - 1 \le 0 $$

Ranges of variables are $0.005 \le {\text{Sp}}_{1} \le 2.00,0.25 \le {\text{Sp}}_{2} \le 1.3,2.00 \le {\text{Sp}}_{3} \le 1$.

#### Multi-disk clutch brake (discrete variables)

**Fig. 29:**
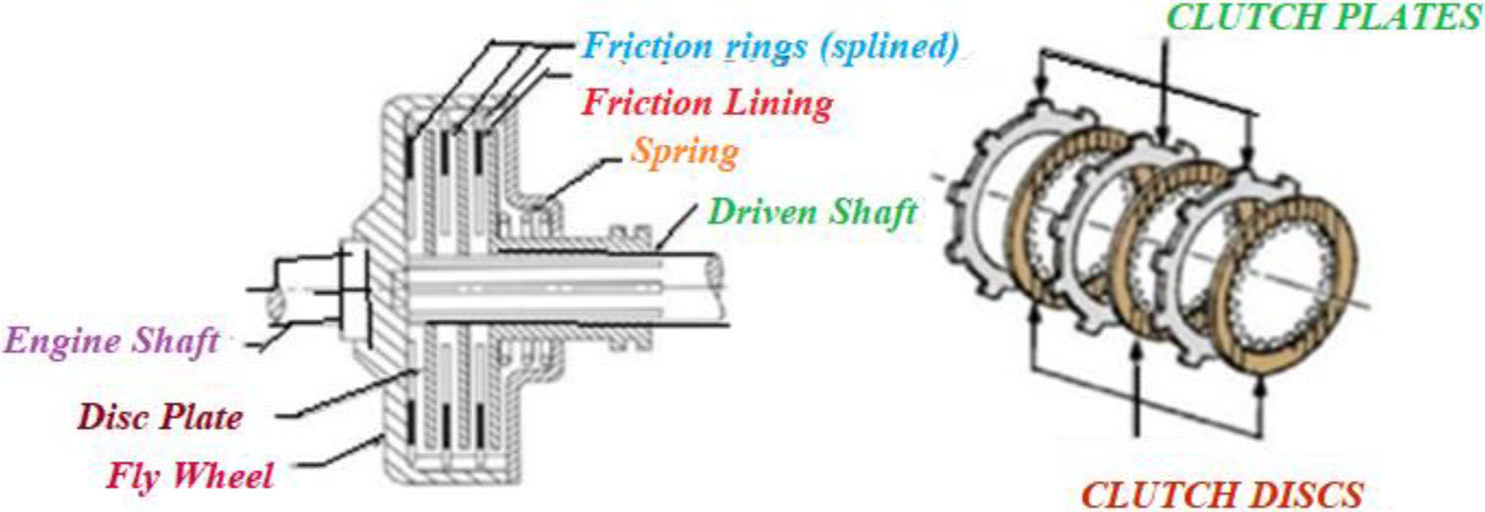
Multiple clutch brake design

The crucial technological challenge is presented in Fig. 29 is the multi-disk clutch brake design difficulty [194]. The target is to minimize or maximize weight; anyways it involves five discrete variables: friction surface number (*F*_sn_), disc thickness (*D*_Th_), radius of outer surface (*O*_sr_), actuating force form (*F*_ac_), and radius of inner surface (*I*_sr_). Equations (31) to (34) describe the equations for this problem. Table 29 measures the outcomes of hSMA-SA with other methods, demonstrating that hSMA-SA feat known approaches in view of reaching optimal fitness. The design is provided with mathematical equations as follows:

**Table 29:** Comparison of hSMA-SA results for multidisc clutch optimization design with known techniques

Competitive techniques	Optimal values for variables					Optimum fitness
	× 1	× 2	× 3	× 4	× 5	
Proposed hSMA-SA	69.99997189	90	1.5	999.9999999	2.312785172	0.389654341
HHO [78]	69.999999	90.00	1.00	1000.00	2.312781994	0.259768993
TLBO [50]	70	90	3	810	1	0.3136566
WCA [187]	70.00	90.00	1.00	910.000	3.00	0.313656
HHO-PS [50]	76.594	96.59401	1.5	1000	2.13829	0.389653
MBFPA [195]	70	90	1	600	2	0.235242457900804
PVS [188]	70	90	1	980	3	0.31366
HHO-SCA [54]	70	90	2.312785	1000	1.5	0.389653842
NSGA-II	70	90	3	1000	1.5	0.4704
MADE [54]	70.00	90	3	810	1	0.3136566

The mathematics is provided:

31 $$ f\left( {O_{{{\text{sr}}}} ,I_{{{\text{sr}}}} ,F_{{{\text{sn}}}} ,D_{{{\text{Th}}}} } \right) = \pi {\text{Th}}\gamma \left( {O_{{{\text{sr}}}}^{2} - I_{{{\text{sr}}}}^{2} } \right)\left( {F_{{{\text{sn}}}} + 1} \right) $$

where

$$ I_{{{\text{sr}}}} \in 60,61,62 \ldots 80;\quad O_{{{\text{sr}}}} \in 90,91, \ldots 110;\quad D_{{{\text{Th}}}} \in 1,1.5,2,2.5,3;\quad F_{{{\text{ac}}}} \in 600,610,620,1000;\quad F_{{{\text{sn}}}} \in 2,3,4,5,6,7,8,9. $$

Subjected to

32 $$ cb_{1} = D_{0} - D_{{{\text{in}}}} - \Delta D \ge 0. $$

32a $$ cb_{2} = L_{{{\text{MAX}}}} - (S_{f} + 1)({\text{Th}} + \alpha ) \ge 0 $$

32b $$ cb_{3} = {\text{PM}}_{{{\text{MAX}}}} - {\text{PM}}_{\pi } \ge 0 $$

32c $$ cb_{4} = {\text{PM}}_{{{\text{MAX}}}} Z_{{{\text{MAX}}}} + {\text{PM}}_{\pi } Z_{{{\text{SR}}}} \ge 0 $$

32d $$ cb_{5} = Z_{{{\text{SR}}_{{{\text{MAX}}}} }} - Z_{{{\text{SR}}}} \ge 0 $$

32e $$ cb_{6} = t_{{{\text{MAX}}}} - t \ge 0 $$

32f $$ cb_{7} = {\text{RC}}_{h} - {\text{RC}}_{f} \ge 0 $$

$$ cb_{8} = t \ge 0 $$

Here, $${\text{PM}}_{\pi } = \frac{{F_{{{\text{ac}}}} }}{{\Pi \left( {D_{0}^{2} - D_{{{\text{in}}}}^{2} } \right)}}$$

$$ Z_{{{\text{SR}}}} = \frac{{2\pi n\left( {D_{0}^{3} - D_{{{\text{in}}}}^{3} } \right)}}{{90\left( {D_{0}^{2} - D_{{{\text{in}}}}^{2} } \right)}} $$

$$ t = \frac{{i_{x} \pi n}}{{30\left( {{\text{RC}}_{h} + {\text{RC}}_{f} } \right)}}. $$

## Conclusion

**Fig. 30:**
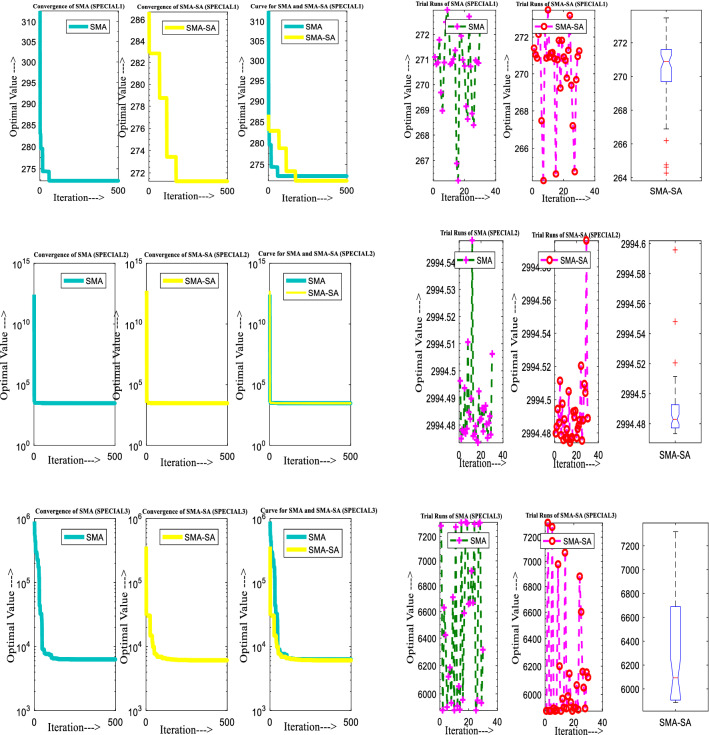

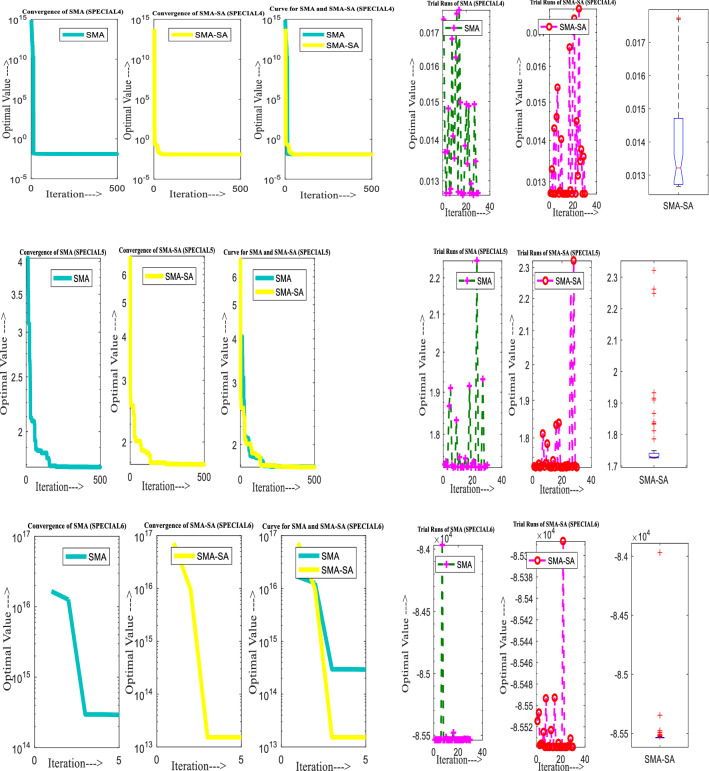

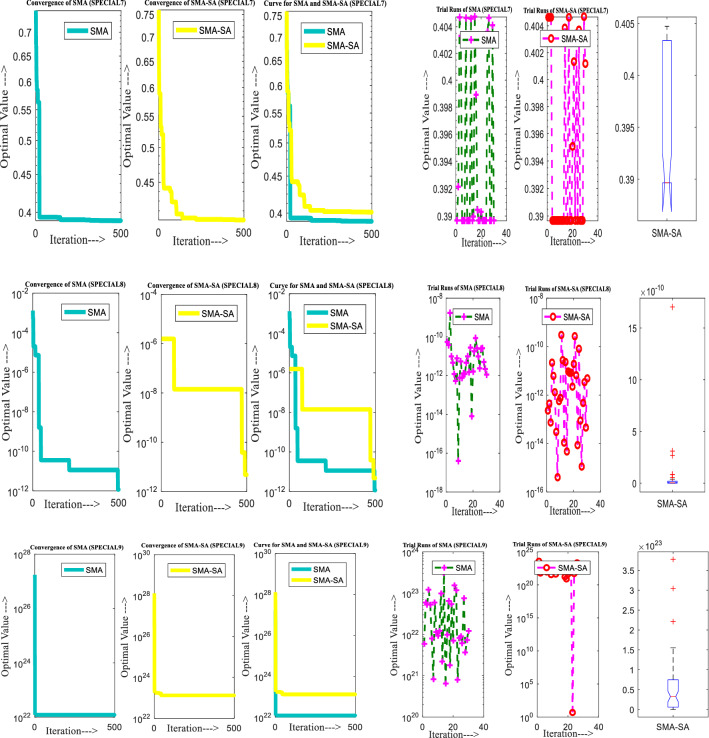

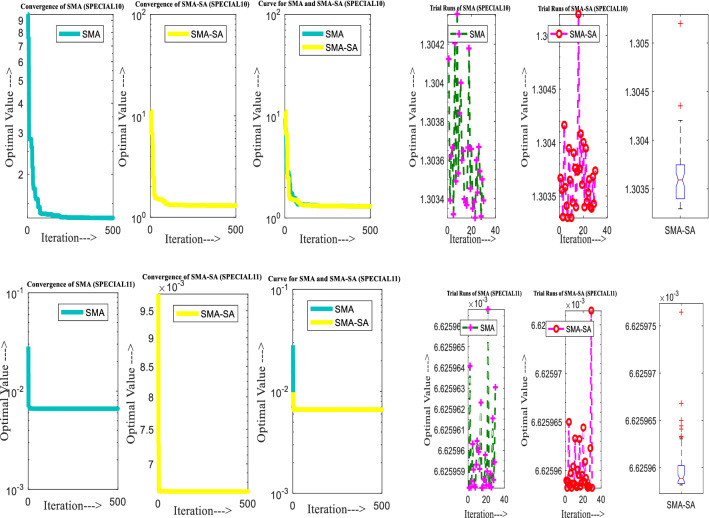
Convergence curve and trial run for special engineering functions with SMA and hSMA-SA

In this work of research, a combination of two optimizers has been fruitfully launched a hybridized optimizer named slime mould-simulated annealing algorithm, which relies on the character of slime mould and enacts the uniqueness of the phase of oscillation (Fig. 30). In the global search region, it uses adaptive weights for wave propagation to identify the best solution. The newly created approach has been examined for a variety of 11 interdisciplinary design problems and traditional benchmark optimization challenges, involving 6—unimodal, 5—multimodal, and 5—fixed-dimension benchmark problems. It has been empirically found that the approach is effective to find the solution inside the global search space after testing the competence of the suggested techniques for typical benchmarks and interdisciplinary engineering design issues. It has been advised that the suggested hybrid optimizer be collectively approved to crack tough special engineering design tasks in the global search space depending on practical findings and comparison study with known approaches. Furthermore, these hybrid variations may be used to tackle the actual power system’s multi-area economic load dispatch problem. The proposed algorithm is taking too much time for high dimensions objective function and going out of memory for 140 units economic load dispatch problem. Hence, the computational capacity of the suggested algorithm is slow for higher dimension benchmark problems. The proposed algorithm proved in obtaining influential and optimal solutions. In the outlook, the proposed SMA variant may be appreciably used to find solutions for different types of engineering and design optimization problems also single-area and multi-area economic load dispatch, generation scheduling problem, and auto-generation control issues of practical power systems; it may be utilized to find solutions for power dispatch issues incorporating PEVs, BEVs, and renewable energy sources.
